# Are Open Science instructions targeted to ecologists and evolutionary biologists sufficient? A literature review of guidelines and journal data policies

**DOI:** 10.1002/ece3.11698

**Published:** 2024-07-10

**Authors:** Elina Koivisto, Elina Mäntylä

**Affiliations:** ^1^ Section of Ecology, Department of Biology University of Turku Turku Finland; ^2^ Present address: Federation of Finnish Learned Societies (TSV) Helsinki Finland

**Keywords:** data policy, data sharing, FAIR, guideline, Open Science

## Abstract

Open science (OS) awareness and skills are increasingly becoming an essential part of everyday scientific work as e.g., many journals require authors to share data. However, following an OS workflow can seem challenging at first. Thus, instructions by journals and other guidelines are important. But how comprehensive are they in the field of ecology and evolutionary biology (Ecol Evol)? To find this out, we reviewed 20 published OS guideline articles aimed for ecologists or evolutionary biologists, together with the data policies of 17 Ecol Evol journals to chart the current landscape of OS guidelines in the field, find potential gaps, identify field‐specific barriers for OS and discuss solutions to overcome these challenges. We found that many of the guideline articles covered similar topics, despite being written for a narrow field or specific target audience. Likewise, many of the guideline articles mentioned similar obstacles that could hinder or postpone a transition to open data sharing. Thus, there could be a need for a more widely known, general OS guideline for Ecol Evol. Following the same guideline could also enhance the uniformity of the OS practices carried on in the field. However, some topics, like long‐term experiments and physical samples, were mentioned surprisingly seldom, although they are typical issues in Ecol Evol. Of the journals, 15 out of 17 expected or at least encouraged data sharing either for all articles or under specific conditions, e.g. for registered reports and 10 of those required data sharing at the submission phase. The coverage of journal data policies varied greatly between journals, from practically non‐existing to very extensive. As journals can contribute greatly by leading the way and making open data useful, we recommend that the publishers and journals would invest in clear and comprehensive data policies and instructions for authors.

## Graphical abstract

Open Science (OS) awareness and skills are increasingly becoming an essential part of everyday scientific work. We reviewed OS guidelines given to ecologists and evolutionary biologists. We also reviewed data policies and instructions of the top journals of this field of science.

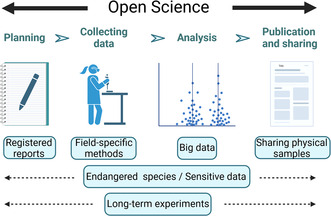

## Abstrakti

Avoimen tieteen ymmärrys ja taitojen hallinta on yhä tärkeämpi osa tutkijan arkea, sillä esimerkiksi monet tieteelliset lehdet odottavat aineiston avointa jakamista. Avoimen tieteen työtapojen noudattaminen voi kuitenkin tuntua alkuun haastavalta, minkä vuoksi esimerkiksi tieteellisten lehtien ja muiden tahojen laatimat ohjeet ovat tärkeitä. Mutta kuinka kattavia ne ovat ekologian ja evoluutiobiologian alalla? Kävimme läpi 20 julkaistua ekologeille tai evoluutiobiologeille suunnattua avoimen tieteen ohjeistusta sekä 17 ekologian ja evoluutiobiologian tieteellisen lehden datakäytännöt, tarkoituksenamme kartoittaa alojen avoimen tieteen ohjeiden nykytilaa, löytää mahdollisia puutteita, tunnistaa alakohtaisia esteitä avoimen tieteen käytäntöjen toteutumiselle sekä keskustella ratkaisuista, joilla nämä haasteet voitaisiin ratkaista. Havaitsimme, että monet ohjeistukset käsittelivät samankaltaisia aiheita, vaikka ne oli tarkoitettu kapealle erityisalalle tai suunnattu hyvin rajoitetulle kohderyhmälle. Samoin monissa ohjeistuksissa mainittiin samankaltaisia aineistojen avoimen jakamisen hidastamista tai estämistä aiheuttavia haasteita. Toiset aiheet, kuten pitkäaikaiskokeet ja fyysiset näytteet, sen sijaan mainittiin yllättävän harvoin, vaikka niissä on tyypillisiä ekologian ja evoluutiobiologian alojen haasteita. Tieteellisistä lehdistä 15:ssä 17:sta vaadittiin tai vähintään kannustettiin jakamaan aineisto avoimesti joko kaikkien artikkelien osalta tai tietyin edellytyksin, esim. rekisteröityjen tutkimusraporttien osalta. Lisäksi 10 näistä lehdistä edellytti aineiston avointa jakamista jo submittointivaiheessa. Tieteellisten lehtien aineisto‐ohjeiden kattavuus vaihteli suuresti lehtien välillä, käytännössä olemattomasta hyvin laajaan. Koska tieteellisillä lehdillä on suuri vaikutusvalta avoimen tieteen käytäntöjen edistämiseen, suosittelemme kustantajia ja lehtiä panostamaan selkeisiin ja kattaviin aineistolinjauksiin ja ohjeistuksiin.

## 1 INTRODUCTION

Open science (OS) is an international movement, which can be seen as an effort to make scientific research (including publications, data, physical samples and software) and its dissemination accessible to all levels of society, amateur or professional. OS can also be seen as a wider construction including aspects of inclusivity and equality. For example, the UNESCO Recommendation on OS provides an international framework for OS policy and practice that recognises disciplinary and regional differences in OS perspectives (https://www.unesco.org/en/open‐science/about). It considers academic freedom, gender‐transformative approaches and the specific challenges of scientists and other OS actors in different countries and in particular in developing countries and contributes to reducing the digital, technological and knowledge divides existing between and within countries.

OS also has several core principles that are used to define the level of openness of the research (https://www.unesco.org/en/open‐science/about). FAIR (Findable, Accessible, Interoperable, Re‐usable) is one of the most common and best‐known principles (Wilkinson et al., 2016). CARE (Collective benefit, Authority to control, Responsibility, Ethics) is one of the newer principles. CARE principles were created in 2019 by the International Indigenous Data Sovereignty Interest Group. Its goal is to settle, throughout the data lifecycle, the rights and interests of indigenous peoples in their data (Carroll et al., 2021). Together with all OS principles researchers can show the open and transparent origin and future of their research.

While OS enhances equity by allowing, for example, access to scientific articles to a wider audience regardless of e.g. their financial status or workplace, it also benefits the authors (McKiernan et al., 2016). For example, Clark et al. (2024) studied the benefits of open access (OA) publishing across various sub‐fields in biology and found a citation advantage for OA articles, meaning that OA outputs receive more citations as compared to non‐OA outputs. Similarly, Colavizza et al. (2024) found that releasing a publication as a preprint correlates with a significant positive citation advantage and that sharing data in an online repository correlates with a smaller yet still positive citation advantage.

Publishing OA is often not just a choice made by authors but a requirement of a funding body. For example, when applying for Horizon Europe funding, researchers are expected to include in the proposal how the project will comply with the mandatory OS practices, which ‘refers to open access to publications and open access to FAIR data, according to the principle ‘as open as possible, as closed as necessary” (https://rea.ec.europa.eu/open‐science_en). In addition, journals are increasingly starting to require authors to share their data together with the submitted manuscript. As Borgman and Brand (2024) aptly put it, ‘the current state of open data in scholarly publishing is in transition from “nice to have” to “need to have”’. Being aware of and being able to implement open research practices is therefore becoming increasingly fundamental to researchers.

Preparing data for sharing can, however, be time‐consuming, laborious and challenging, especially if the researcher responsible for the preparation is lacking appropriate resources, guidelines or support. There can also be uncertainty of the suitable data format, or how the metadata should be formulated or which repository to use. Kim (2021) found 354 repositories used to store data for ecological research, so deciding which one to use can be an overwhelming task. In a survey conducted by SpringerNature, Stuart et al. (2018) found that the main challenge to data sharing was identified by respondents as ‘Organizing data in a presentable and useful way’ (46%), followed by ‘Unsure about copyright and licensing’ (37%), ‘Not knowing which repository to use’ (33%), ‘Lack of time to deposit data’ (26%) and ‘Costs of sharing data’ (19%). Likewise, in their review for biological sciences, Gomes et al. (2022) grouped the common reasons for the failure to adopt open data and code practices into three broad categories: knowledge barriers, reuse concerns and career incentives.

Despite these barriers, Tedersoo et al. (2021) who evaluated data availability in research articles across nine disciplines (including ecology) in the journals Nature and Science, found that data availability in top scientific journals differs strongly by discipline, but it is improving in most research fields. However, merely sharing the data is not adequate if it cannot be used. Purgar et al. (2022) quantified the research waste in ecology and came to a shocking conclusion, that only 11%–18% of conducted ecological research reaches its full informative value. Reasons for research waste are numerous (e.g. poorly planned studies, unpublished results) but unpublished or unusable published data (e.g. data shared without proper metadata) are also playing a major role.

Different disciplines can have different traditions with their research methods and special challenges related in particular to data, which can affect the attitude towards OS and willingness to share data and other resources. Ecology and evolutionary biology (hereafter Ecol Evol) are typical data‐driven fields of research, in which the collection of research data can thus be also seen as a currency. Poisot et al. (2019) claimed that ecologists are either not aware or encouraged enough to use open, programmatically searchable, structured, specialised repositories for ecological data. As one key issue they identified that many domains in ecology ‘lack well‐established, appropriate and specific standards’. According to them, the lack of established and specific standards is one reason which can discourage ecologists to share their research data in a well‐structured and machine‐readable way (Poisot et al., 2019).

**FIGURE 1:**
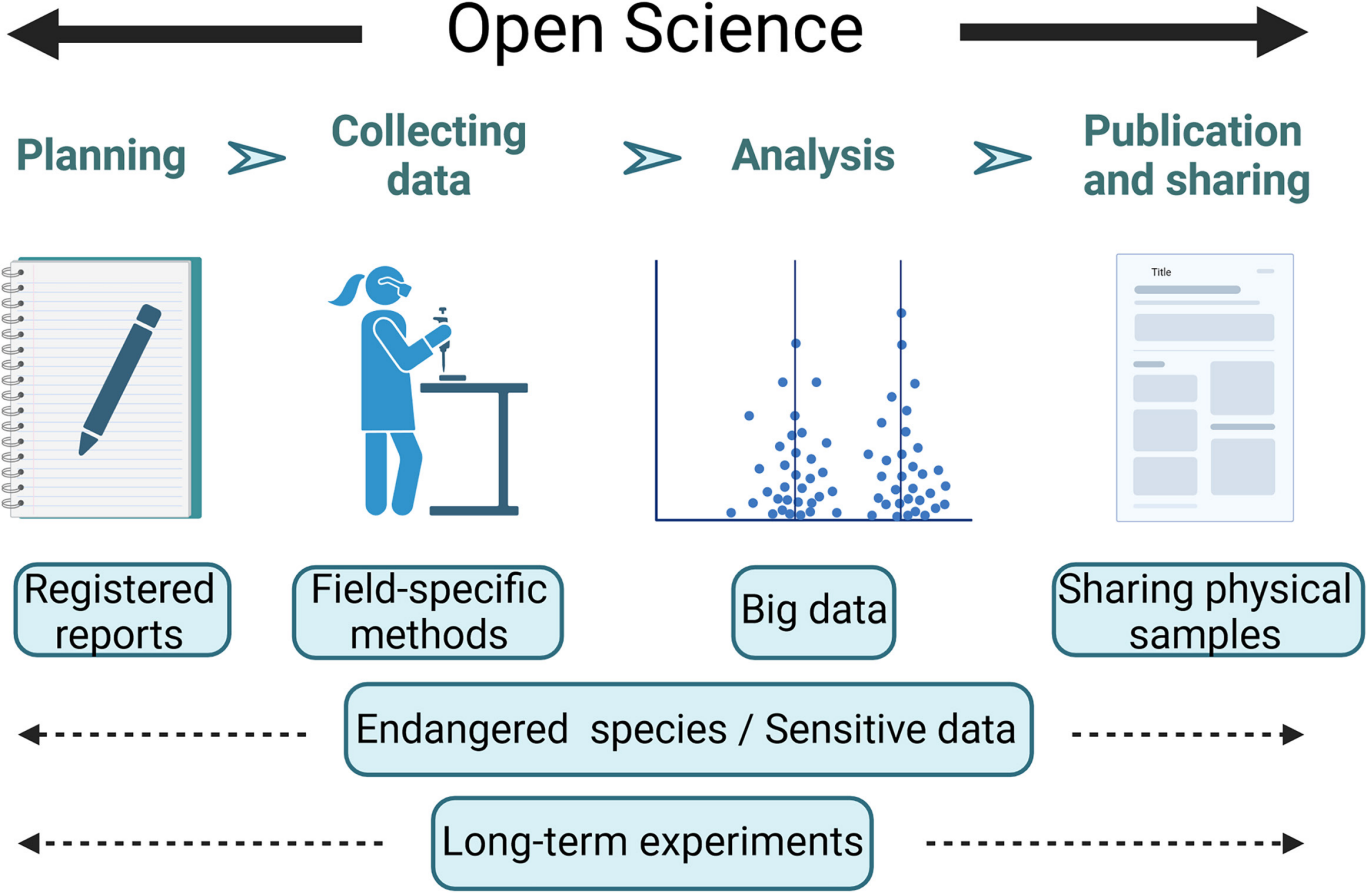
Open Science (OS) is used in all stages of the research project: planning, data collection, analyses, and publication and sharing the acquired results. This figure presents some of the ecology and evolutionary biology specific, OS‐related topics discussed in this study. Created with BioRender.com.

As the research conducted in the fields of Ecol Evol can be very different from each other, also the challenges in OS can differ a lot even within the same discipline. The challenges can be different with sensitive DNA samples, thousands of trail camera photos or field data collected by dozens of researchers. Common data‐related OS issues in ecology include, for example, long‐term datasets, sensitive data on locations of rare species and large spatial datasets (Figure 1).

If the OS guidelines are too general, they might not serve the researchers searching answers to very special questions regarding their own datasets. Thus, field‐specific guidelines are important. But how comprehensive are they in the fields of Ecol Evol?

To answer this, we reviewed altogether 20 OS guidelines aimed for Ecol Evol researchers, together with the data policies of 17 top Ecol Evol journals. Our aim was to (1) chart the current landscape of OS guidelines for Ecol Evol, (2) identify potential existing gaps in guidelines (especially in relation to journal data policies) and (3) identify potential Ecol Evol special issues and barriers for OS and discuss solutions.

## 2 MATERIALS AND METHODS

The inspiration for this study came from the material collected for the ROSiE (Responsible OS in Europe) project's deliverable ‘ROSiE Field‐specific Guidelines on Responsible Open Science’ (part ‘Natural Sciences’; Rochambeau et al., 2024). During the collecting phase it was noticed that compared to some other fields, there was a high number of OS guidelines aimed for Ecol Evol. As we found that interesting, we wanted to perform a literature review on the guidelines on the Ecol Evol viewpoint. The material for ROSiE guidelines was collected already earlier in 2023 and we wanted to include possible guidelines published after the ROSiE collection (late 2023 and early 2024). Also, we wanted to make sure that no relevant guidelines were missed during the ROSiE collection.

The first step in conducting this study was thus to list the publicly available resources potentially related to OS guidelines in Ecol Evol, or closely related fields into one spreadsheets document. The methodology for finding those resources included the following:

- Using the resources collected in the ROSiE (Responsible OS in Europe) project for Field‐specific Guidelines (part ‘Natural Sciences’; Rochambeau et al., 2024)
- A search conducted in Google Scholar using the following search terms: (ecolog* OR biolog*) AND ‘OS’ AND (guideline* OR polic*)
- Citations from already acquired articles
- Previous knowledge of the authors

We want to emphasise that the search was not aimed to be exhaustive, nor was our aim to conduct a systematic review. The list of search terms is partly based on the keywords found in the originally existing resources (e.g. the guidelines found for the ROSiE project), partly on our previous knowledge and partly on testing combinations of search terms. We wanted to find as many relevant guidelines as possible while not making the literature search too heavy.

We are aware that there are also high‐quality OS guidelines available for example in academic publishers' or ecological societies' online resources (e.g. https://www.britishecologicalsociety.org/publications/better‐science/). These were, however, not included in this study, as we wanted to limit our search only to peer‐reviewed, published articles as they are not restricted to any specific journal or publisher, should have similar standards for offered guidelines and are thus more general in style.

We found in total 20 published articles with OS guidelines for Ecol Evol researchers by January 2024. In the next phase we read through all these guidelines and collected the following information for each one:

- name of first author, publication year, journal, target audience and specific topic

In addition, we marked down if the resource mentioned the following terms (yes/no). These terms were chosen based on our previous knowledge and our preliminary qualitative analysis of the terms appearing regularly in the found guidelines. We aimed for a list of general terms that are valid for describing different but common aspects of OS.

- list of terms: big data, CARE, code, data management plan, electronic laboratory notebook, FAIR, licences, long‐term experiments, metadata, methods, OS communities, open‐source software, persistent identifiers (PIDs), physical samples/material, preprint, preregistration, registered reports, repositories, sensitive data, tools

We also reviewed the data policies of the top 10 journals in ecology and the top 10 journals in evolutionary biology, listed by Cornell University Library based on 2021's impact factor (https://guides.library.cornell.edu/c.php?g=150193&p=2508503). Since four of the journals occurred in both lists, we ended up having 16 journals. In addition, we also reviewed the data policy of Ecology and Evolution, as it was our target journal. The final number of journals was thus 17.

We checked the journal websites to review the data policy (see Appendix S1 for the actual text of the data policies retrieved in May 2024). We went through all the relevant parts (e.g. instructions for authors, for authors, author guidelines, for submission, author resource centre) to find guidance on how to handle data and code. If we did not find any data‐related guidance in these sections, we assumed that the journal does not have any.

Afterwards we divided the journals to three categories (1, 2, 3) based on the extent of the data guidelines, category one being the narrowest and category three the most comprehensive. The criteria for categories 1, 2 and 3 were defined as follows:

1. The journal has no obvious data policy to be found on their website.
2. The journal has either a separate data policy or a clear data section in their ‘instructions for authors’ or similar and the journal *requires or encourages* data sharing *after acceptance*.
3. The journal has either a separate data policy or a clear data section in their ‘instructions for authors’ or similar and the journal *requires* data sharing *on submission*.

We also checked the following points:

- Data‐sharing policy (e.g. required or encouraged)
- if data sharing is required/encouraged, is it required/encouraged at submission or after acceptance
- is metadata, README file or equivalent information required
- does the journal ask for data availability statement or equivalent information
- does the journal provide suggestions for repositories
- code sharing policy (e.g. required or encouraged)

## 3 RESULTS

### 3.1 Guideline articles

Of the 20 published articles with guidelines for ecologists and evolutionary biologists (Table 1), some were for a more general audience and some were for a specific niche, such as hydrologists (Hall et al., 2022) or palaeoecologists (Flantua et al., 2023). Despite these different original views, there were a lot of similar guidelines and recommendations (Appendix: Table S2).

**TABLE 1:** The OS guideline articles aimed for Ecol Evol researchers of different subfields reviewed for this study.

First author	Journal	Year	Target audience	Specific topic
Hampton et al.	Ecosphere	2015		
Levin et al.	Science, Technology, & Human Values	2017		
Ihle et al.	Behavioural Ecology	2017	Behavioural ecologists	Transparency
Scotson et al.	Remote Sensing in Ecology and Conservation	2017	Wildlife ecologists	Camera trap data
Powers et al.	Ecological Applications	2019	Ecology	Reproducibility
Zipper et al.	Water Resources Research	2019	Water science	Data privacy
Mustaphi et al.	Quaternary Geochronology	2019	Geochronology	Sediment chronologies
Wittman et al.	Journal of Insect Science	2020	Entomologists	Replication
Geange et al.	Ecology and Evolution	2020		Field courses
Kühl et al.	One Earth	2020		Biodiversity monitoring
Brock et al.	Integrated Environmental Assessment and Management	2021	Environmental risk assessment	
Diederich et al.	PLOS Biology	2022	Animal research	
Hall et al.	Hydrology and Earth System Sciences	2022	Hydrologists	
Muñoz‐Tamayo et al.	PNAS Nexus	2022	Animal scientists	
Pourret et al.	Frontiers in Research Metrics and Analytics	2022		Inclusive Metrics
Jenkins et al.	Ecology and Evolution	2023		Reproducibility
Bertram et al.	Current Biology	2023		
Flantua et al.	Global Ecology and Biogeography	2023	Palaeoecologists	Fossil pollen data
Bubnicki et al.	Remote Sensing in Ecology and Conservation	2024		Camera trap data
Abdill et al.	ArXiv	2024		Code sharing

*Note*: The table lists the surname of the first author, journal name, publication year and if target audience or specific topic were defined.

Almost all mentioned OS in relation to repositories (100%), metadata (85%), tools (85%), methods (85%), code (80%) and open‐source software (75%). The authors often listed repositories used in that field of science (e.g. Re3data, FAIRsharing, OS Framework, figshare, Dryad, Zenodo). About metadata it was usually told that it is needed for reproducible science and researchers should use common metadata standards of that field (e.g. Ecological Metadata Language (EML), NetCDF). Many articles had tables or lists of useful tools to make transition to OS easier (e.g. Bertram et al., 2023; Hampton et al., 2015). The guidelines for OS methods emphasised the use of common or standardised methodology and to report the used methods in detail. The sharing of used code is explained in detail in a recent article (Abdill et al., 2024). The instructions for open‐source software were often combined with open code. The use of non‐proprietary software was the most common guideline.

About half of the guideline articles mentioned FAIR principles (65%), data management plans (60%), PIDs (60%), licences (50%), sensitive data (50%), electronic laboratory notebooks (45%), OS communities (45%) and preprints (40%). The FAIR principles were defined in 2016, so it is understandable that the older articles do not mention those (Wilkinson et al., 2016). Of PIDs the only one mentioned was DOI, although there also other PIDs available. Use of licences, especially CC licences, was often combined with information about PIDs. Sensitive data could have been mentioned more often, as in biology it is not only about endangered species and personal details but also trade, proprietary and government information. Electronic laboratory notebooks could be used more often to ensure smooth transition of research protocol and methods to the next phases in the research project timeline. Many guidelines pointed out that the OS is best distributed through local or field specific OS communities (Bertram et al., 2023; Hall et al., 2022; Levin & Leonelli, 2017).

The topics that were mentioned least often were the following: long‐term experiments (35%), preregistration (30%), registered reports (25%), physical samples/research material (25%), big data (20%) and CARE (10%). Some of these least mentioned topics are quite new in Ecol Evol, such as preregistration, registered reports and CARE. Preregistration is used to specify the research plan in advance of the study and submit it to a registry (https://www.cos.io/initiatives/prereg). A Registered Report (RR) is an original research article which undergoes a two‐stage peer review process (https://www.cos.io/initiatives/registered‐reports). First review is of introduction, methods, proposed analyses and pilot data (stage 1). The next step is ‘In principle acceptance’ before data collection commences. Once the study is complete, the authors finalise the article to include results and discussion (stage 2). The manuscript is peer‐reviewed again and can be accepted or rejected. Journals can have specific data policies for RRs. Researchers are slowly realising the OS aspects related to more traditional topics of long‐term experiments, physical samples and big data.

### 3.2 Journal data policies

Of the 17 journals of which we checked data policies, two (12%) were ranked to category 1 (no obvious data policy to be found), seven (41%) to category 2 (data policy requires/encourages data sharing after acceptance) and eight (47%) to category 3 (data policy requires data sharing on submission, Table 2). Of the 17 journals, 12 required data sharing, one required data sharing for RRs, one required data sharing ‘when ethically possible’ and one encouraged data sharing. Of those 15 journals, which required or encouraged data sharing, 10 required data sharing at submission phase and five after acceptance (Table 2). Seven journals required to include metadata, README file or similar information and additional two journals recommended or encouraged to include metadata or README file.

**TABLE 2:** The journal data policies reviewed for this study.

Number	Name of the journal	Data policy score (1–3)	Data availability policy	Link to data policy/author guidelines/similar	If data is required, when?	Data availability statement	Metadata/README/similar	Suggestions for repositories	Policy for code
1	Annual Review of Ecology, Evolution and Systematics	1	No	https://www.annualreviews.org/page/authors/editorial‐policies#authorship	NS	No	NS	NS	NS
2	Cladistics	3	Required	https://authorservices.wiley.com/author‐resources/Journal‐Authors/open‐access/data‐sharing‐citation/data‐sharing‐policy.html	On submission	Yes	Encouraged	Yes	Encouraged
3	Ecological Monographs	2	Required	https://www.esa.org/publications/data‐policy/	After acceptance	Yes	Required	Yes	Required
4	Ecology and Evolution	3	Required	https://onlinelibrary.wiley.com/page/journal/20457758/homepage/forauthors.html	On submission	Yes	Required	Yes	Required
5	Ecology Letters	3	Required	https://onlinelibrary.wiley.com/page/journal/14610248/homepage/forauthors.html#data‐availability	On submission	Yes	Required	Yes	Required
6	Evolution Letters	3	Required (when ethically possible)	https://academic.oup.com/evlett/pages/general‐instructions#Availability_of_data_and_materials	On submission	Yes	No	Yes	Required (when ethically possible)
7	Frontiers in Ecology and the Environment	2	Required	https://www.esa.org/publications/data‐policy/	After acceptance	Yes	Required	Yes	Required
8	Global Change Biology	3	Required	https://onlinelibrary.wiley.com/page/journal/13652486/homepage/forauthors.html	On submission	Yes	No	Yes	Encouraged
9	Methods in Ecology and Evolution	3	Required	https://besjournals.onlinelibrary.wiley.com/hub/journal/2041210X/author‐guidelines	on submission	Yes	No	Yes	Required
10	Molecular Biology and Evolution	2	Required	https://academic.oup.com/mbe/pages/General_Author_Guidelines	After acceptance	Yes	Recommended	Yes	No
11	Molecular Ecology	3	Required	https://onlinelibrary.wiley.com/page/journal/1365294x/homepage/forauthors.html	on submission	Yes	Required	Yes	Encouraged
12	Molecular Ecology Resources	3	Required	https://onlinelibrary.wiley.com/page/journal/17550998/homepage/forauthors.html	on submission	Yes	Required	Yes	Encouraged
13	Nature Ecology & Evolution	2	Required (for RR)	https://www.nature.com/natecolevol/submission‐guidelines/registeredreports	After acceptance	Yes for RR	No	No	Required for RR
14	Proceedings of the Royal Society B‐Biological Sciences	3	Required	https://royalsocietypublishing.org/rspb/for‐authors#question6	On submission	Yes	Required	Yes	Required
15	Systematic Biology	3	Required	https://academic.oup.com/sysbio/pages/General_Instructions	On submission	Yes	No	Yes	No
16	The ISME Journal	2	Encouraged	https://www.springernature.com/gp/authors/research‐data‐policy	After acceptance	Yes	No	Yes	Reviewers can request
17	Trends in Ecology and Evolution	1	No	https://www.cell.com/trends/ecology‐evolution/authors	NS	No	No	NS	NS

*Note*: The table lists for each journal the data policy score (1–3, please see Section 2 for details), data availability policy, link to data policy, author guidelines containing data policy or similar information (if available), at what state of the publication process the data are required (if required), if the journal asks for a data availability statement, if the journal asks for metadata/README file or similar information, if the journal gives suggestions for repositories and what is the journal policy for code. Journals are listed in alphabetical order. Please see Appendix S1 for the actual text of the data policies (retrieved in May 2024).

Abbreviations: NS, not significant; RR, registered report.

Altogether 14 journals asked for a data availability statement or similar and an additional one asked it for RRs. Similarly, 14 journals gave suggestions for suitable repositories (Table 2). Six journals required code sharing, one required it for RRs and one required code sharing ‘when ethically possible’. In addition, four journals encouraged code sharing and an additional one had a note that reviewers have a right to require the code.

### 3.3 Issues mentioned

The same 20 published articles offering OS guidelines also mentioned issues that could hinder or slow transition to OS. Some articles had a specific table listing these issues or problems (Hall et al., 2022; Scotson et al., 2017). Here are listed the most common ones based on categories presented by Gomes et al. (2022):

Reuse concerns

- sensitive research data (e.g. research participants, endangered species)
- fear of data or code could be used inappropriately
- a variety of reporting styles can lead to data being effectively unavailable for further scientific enquiry (e.g. location, date information)
- restrictions on practising OS due to public and private institutional rules, national policies and data sovereignty and governance of stakeholders and collaborators

Disincentives

- preprint publications could be of lesser quality due to a lack of proper peer review
- no formal credit for outputs beyond articles
- if the research process cannot be transparent, then reliability and reproducibility are difficult to guarantee
- effort and costs of publishing datasets, engaging the public and communicating findings
- lack of capacity, high turnover of staff and a failure to use standardised protocols
- open research can expose researchers to new avenues for harassment and suppression
- lack of and/or limited funds to afford the high cost of open access publishing

Knowledge barriers

- time required to invest in learning the use of OS tools
- lack of institutionalised incentives and training opportunities
- in smaller or more remote institutes there can be lack of access to technology, software, training and materials to facilitate good data management

## 4 DISCUSSION

We reviewed altogether 20 published OS guideline articles aimed for ecologists or evolutionary biologists, together with the data policies of 17 ecology or evolutionary biology journals to chart the current landscape of OS guidelines in Ecol Evol. We wanted to identify potential existing gaps in guidelines (especially in relation to journal data policies) and issues and barriers for OS typical for Ecol Evol.

We found that many of the instructions and guidelines offered in articles were similar, as were the issues these articles mentioned that could hinder or postpone transition to more OS data sharing. We did, however, find a few potential gaps in the instructions, such as long‐term experiments and physical research material. The coverage of journal data policies varied greatly between journals, from practically non‐existent to very extensive. We discuss our findings in detail below, together with possible solutions for the emerged issues.

### 4.1 Guideline articles

Already for several years good data management has relied on FAIR principles (Wilkinson et al., 2016). These four foundational principles (findability, accessibility, interoperability and reproducibility) should be adopted as much as possible in different stages of the research process: planning, data collection, analyses and reporting. Nowadays CARE principles (collective benefit, authority to control, responsibility, ethics) for Indigenous Data Governance are often combined with FAIR principles in OS (Carroll et al., 2021). CARE is not yet that common in guidelines, but it can be expected to be more visible in the future as responsible data management is seen as a core value (van der Aalst et al., 2017). And handling of sensitive data is seen as a crucial element of good data management. Often ecological and biological field experiments are placed in the global south or other remote locations. It would be crucial to cooperate with local researchers and train interested locals to assist with experiments using OS principles, such as FAIR and CARE (Jennings et al., 2023).

Several of the articles we examined for OS guidelines had excellent tables of useful tools in different stages of the research (e.g. Diederich et al., 2022; Hall et al., 2022). They give suggestions of open‐source software, how to write transparent code, choose accessible data visualisations and which electronic laboratory notebooks could be useful. Many of the guidelines especially point out the detailed reporting of all used methods; from choosing sampling locations to measurement units and reporting unexpected incidents during data collection.

When preparing the data management plan, it is good to have a clear vision of the workflow. The studied guideline articles include good examples of what the workflow should look like for an open and reproducible research project (e.g. Bertram et al., 2023; Hampton et al., 2015; Wittman & Aukema, 2020).

There are studies showing that articles that were first published as preprints can gain more citations and higher Altmetrics Scores (e.g. Fu & Hughey, 2019). The most used preprint servers for Ecol Evol studies are bioRxiv (https://www.biorxiv.org/) and EcoEvoRxiv (https://ecoevorxiv.org/). Both have detailed instructions on how to submit a preprint article on their websites.

Journals often list suitable repositories where to save the research data used for the published article. This usually requires the author to write a data availability statement. It would be helpful for the author if the journals provided a template for this. Statements like ‘data is available from the author with reasonable request’ are not adequate anymore (Tedersoo et al., 2021). If the data are sensitive or otherwise cannot be shared as whole, the researcher can, for example, share the metadata or anonymise the data. In the current world of OS, it would be best to think first ‘how can I open my data’ and not ‘I cannot open my data’. The authors will get a PID (usually DOI) for their dataset when they share the data at a repository. This DOI can then be used in other locations to show access to the dataset and during sharing to the repository, authors can choose a suitable licence (CC licence; https://creativecommons.org/share‐your‐work/cclicenses/) to specify the level of openness.

For ecology and other biological fields, data management of long‐term experiments, analysing big datasets, correct sharing of information about endangered species and reproducible storing of physical samples are important. Thus, it was somewhat surprising that those topics were not mentioned more often in guidelines. For example, research with endangered species often also includes environmental challenges such as habitat loss, climate change and declining populations. It is important to share these kinds of sensitive data with care that no additional harm is caused to the endangered species or their environment.

With long‐term experiments it can be difficult to share data and results if the data collection is planned to continue. One possibility could be to share at least some of the data regularly, for example, yearly and then use suitable parts of it for publications whenever needed. In addition, data is often used for several studies, so it can be difficult to decide when it can be made available for other interested researchers. Another issue is standardising the research methods if over the years several different researchers have been involved in data collection. Often especially field work requires unique methods that can be challenging to report and share in detail. There are standard methods that can be used but they do not apply for every possible situation. Researchers should try to describe the used methods as clearly as possible to allow reproducible science. One more possible problem with long‐term experiments is that, especially for early‐career researchers, it can take years before any results are ready to be published and data could be shared. This can place these researchers working on long‐term experiments in disadvantage compared to researchers conducting shorter experiments potentially capable of publishing articles in a shorter time interval. It would require a cultural shift and well‐accepted standards in data storage and curation to acknowledge differences in the rate of publishing and sharing results as well as data (Poisot et al., 2019).

The guideline articles had very little information on how to make the physical samples or research material available for future research (e.g. Flantua et al., 2023; Ihle et al., 2017). Physical samples or research material often need special storing conditions such as freezer, ethanol or low humidity, so their open sharing can be challenging. The storing facilities can be expensive, especially if the samples require a lot of space because of their large size or a large quantity. An open registry of the existing samples and their metadata is a prerequisite and a first step for the reuse of the samples.

Systematic bias in the publication process may favour the publication of both positive and significant results, which in turn can cause scientific misconduct such as p‐hacking and fraud (Head et al., 2015). This can lead to ‘reproducibility crisis’, where results are difficult to reproduce by other research groups (Baker, 2016). Preregistration and RRs, which can be one solution to this problem, are already common in some fields of science (Chambers & Tzavella, 2022), but they could become more used also in Ecol Evol (O'Dea et al., 2021). For example, students participating in field courses could remake the traditional project proposals as RRs (Geange et al., 2020).

### 4.2 Journal data policies

Most of the Ecol Evol journals we reviewed had at least some kind of data policy, which was usually placed in the ‘instructions for authors’ or similar section under its own subtitle and was thus easy to find. In some journals, however, it was difficult to know where to find the data policy or if it even exists. The coverage of the policy was found to vary greatly between journals. The scope of the journal naturally affects the contents of the data policy; for example, the journals belonging to the field of molecular biology tended to have detailed instructions, most probably due to the nature of the data in that field of research. In addition, journals that accept RRs tended to have detailed instructions for RRs but not so much for standard articles.

Since we limited our review to cover only the top Ecol Evol journals (based on IF), this might not reflect the state of journal data policies in the fields as whole. However, the top journals are very likely to be the journals to which authors most often submit their work and therefore their data policies are the ones the authors most often encounter. In addition, since a large variation in data policies was already found in this small subsample, the variation in the journals overall could be even larger.

A majority of journals asked for a data availability statement. A data availability statement gives the authors a distinct section where to place all the relevant data‐related information, such as where the data can be found or why the data or parts of it could not be shared. Providing templates for the data availability statement makes it easier for the authors to outline their own statement. For example, if the journal does not approve that data will be available ‘upon a reasonable request’, it should be clearly stated in the instructions.

### 4.3 Issues mentioned

A majority of the issues mentioned in the guideline articles slowing or hindering the use of OS for Ecol Evol researchers were disincentives, such as cost of open access, no formal merit of sharing data or methods, or problems to guarantee reliability and reproducibility. A recent study reported that even though organisations often have OS policies, they rarely have tools to reward scientists about OS activities (Grattarola et al., 2024). There clearly is a need for a culture shift towards OS. Both early‐career and senior researchers could learn from each other in adapting to following OS guidelines.

Especially important for OS practices becoming the new normal would be to provide clear merit for researchers advocating OS (see Grattarola et al., 2024). This needs recognition from different entities such as scientific societies (e.g. Society for Open Reliable and Transparent Ecology and Evolutionary Biology, SORTEE; https://www.sortee.org/; O'Dea et al., 2021). SORTEE aims to promote transparent research practices and foster communities of researchers eager in improving research and institutional incentives in Ecol Evol (O'Dea et al., 2021). But most importantly recognition is needed from influential entities such as journals, funders and policy makers. Indeed, Tedersoo et al. (2021) recommends that to improve data sharing at the time of manuscript acceptance, researchers should be better motivated to release their data with real benefits such as recognition, or bonus points in grant and job applications. Fortunately, there are significant initiatives promoting responsible research assessment, such as CoARA (Coalition for Advancing Research Assessment, https://coara.eu/).

The guideline articles also mentioned several concerns of incorrect or misuse of the shared data. For sensitive data it is possible to share at least the metadata if anonymisation or pseudonymisation are not possible. Misunderstandings in reuse of data and methods can hopefully be diminished with better use of discipline‐specific vocabulary and standards. These concerns can be mitigated by choosing appropriate licences for reuse and writing clear README documents, instructions and metadata. The open dataset should be accompanied with detailed instructions on how to cite it, so that also the original researchers receive the credit they deserve.

There were, however, not that many concerns about knowledge barriers. This could indicate that Ecol Evol researchers do know how to make their research more open, but there are other barriers limiting the sharing. Local OS communities or clubs could be a good solution to advocate the benefits of OS to all researchers, from graduate students to professors. These could be easy‐access situations for researchers to learn and discuss current OS topics. If the OS guidelines and data sharing principles seem overwhelming, researchers can, for example, start by trying if they can reproduce their own results with their code and data and then ask a colleague to do the same before sharing code and data to the whole research community (Popovic et al., 2024). There is already the International Network of OS & Scholarship Communities (INOSC; https://osc‐international.com/) helping to coordinate OS actions globally.

## 5 CONCLUSIONS

Although the guideline articles we found were very practical and thorough, they will not serve the researchers if they are not known or found. The fact that there was a large number of OS guideline articles for Ecol Evol and that many of them contained similar instructions, could signal that the authors of these guidelines were not aware of the already existing, previously published guidelines or did not see them relevant because of a different subfield of research. Alternatively, Ecol Evol researchers could also be exceptionally willing to share their OS knowledge with other researchers. Nevertheless, there could be a need for a more widely known, general OS guideline for Ecol Evol. Following the same guideline could also enhance the uniformity of the OS practices carried on in the field. Another question is what entity could be responsible for sharing such a guideline. Also, as OS is a constantly and fast developing field, it would be beneficial if the guidelines were presented in a living, frequently updated document. The updating should, however, not be the responsibility of individual researchers but rather a task of larger and more stable operators, such as institutions, communities and publishers, just to name a few. In addition, Ecol Evol societies and OS communities could play a key role in the process.

In an agreement with Poisot et al. (2019), we also believe that journals can contribute greatly by leading the way and making open data useful. Poisot et al. (2019) say that this can be done by ‘requiring the deposition of data in appropriate databases with a clear and documented format, which is inspired by research practices, whenever they exist’. Journals do not, however, have to start from scratch, but can use existing guidelines as such or as a template, like Ecology and Evolution has done with Jenkins et al. (2023). We recommend the publishers and the journals to invest in clear and comprehensive data policies and instructions for authors. Unequivocal instructions would reduce the workload for both authors and journal editors.

We also want to highlight the need for investment in data management in general. Authors would benefit from the help of data stewards working in their institutions or data editors working for journals and publishers. It would make the stored and shared data, code and methods better quality and thus reproducible for future generations of researchers.

## AUTHOR CONTRIBUTIONS

**Elina Koivisto:** Conceptualization (equal); data curation (supporting); formal analysis (supporting); investigation (lead); writing – original draft (equal); writing – review and editing (equal). **Elina Mäntylä:** Conceptualization (equal); data curation (lead); formal analysis (lead); investigation (supporting); writing – original draft (equal); writing – review and editing (equal).

## CONFLICT OF INTEREST STATEMENT

The authors declare that there are no competing interests.

## Supporting information


Appendix S1

Table S2

## Data Availability

The data needed to create the main text and tables is presented in Tables 1 and 2 and in the Appendix S1 and Table S2.
