# Supplementary material for: Are Open Science instructions targeted to ecologists and evolutionary biologists sufficient? A literature review of guidelines and journal data policies
Source: Ecol Evol. 2024 Jul 10;14(7):e11698. doi: 10.1002/ece3.11698 (PMC11237169; doi:10.1002/ece3.11698)
Supplement: Supplementary file 1 — Appendix S1 [file ECE3-14-e11698-s001.docx]

Journal data policies

The data policies were retrieved from journal/publisher websites (link included with the policy) during **May 2024**.

[1. Annual Review of Ecology, Evolution, and Systematics](#_eqfrvgdmxuoo)

[2. Cladistics](#_61pv8zz0a57u)

[3. Ecological Monographs](#_kwjceqidy2nu)

[4. Ecology and Evolution](#_5byn3vbxv23n)

[5. Ecology Letters](#_llbpdg47j7ov)

[6. Evolution Letters](#_2k78918d4sx1)

[7. Frontiers in Ecology and the Environment](#_fmb3761pr1mv)

[8. Global Change Biology](#_3x7supwp0i3h)

[9. Methods in Ecology and Evolution](#_a0fcv5avecjk)

[10. Molecular Biology and Evolution](#_itlg1f1ev7n7)

[11. Molecular Ecology](#_w40j0661bkr)

[12. Molecular Ecology Resources](#_h6c2ygjteeyd)

[13. Nature Ecology & Evolution](#_6haz1ja5fz3b)

[14. Proceedings of the Royal Society B-Biological Sciences](#_i9s624b4869y)

[15. Systematic Biology](#_olmo8mz2cf3q)

[16. The ISME Journal](#_bgxoidwyekz9)

[17. Trends in Ecology and Evolution](#_u8zheytpxby)

# 1. Annual Review of Ecology, Evolution, and Systematics

<https://www.annualreviews.org/page/authors/editorial-policies#authorship>

## Data Sharing

In view of the importance of Annual Reviews articles in defining the current state of scientific knowledge, authors should strive to be fair, yet discriminating, in their selection of references. Authors should avoid the inclusion of unpublished materials (including data, code, or unreviewed/unpublished manuscripts). When including unpublished materials in a review, authors must indicate that the material has not been published and include a statement about data availability when appropriate.

# 2. Cladistics

<https://authorservices.wiley.com/author-resources/Journal-Authors/open-access/data-sharing-citation/data-sharing-policy.html>

## Data Sharing and Data Availability

This journal requires data sharing (see below). Review Wiley’s Data Sharing policy where you will be able to see and select the data availability statement that is right for your submission.

<https://authorservices.wiley.com/author-resources/Journal-Authors/open-access/data-sharing-citation/data-sharing-policy.html>

## Wiley’s Data Sharing Policies

Wiley is committed to a more open research landscape, facilitating faster and more effective research discovery by enabling reproducibility and verification of data, methodology and reporting standards. We encourage authors of articles published in our journals to share their research data including, but not limited to: raw data, processed data, software, algorithms, protocols, methods, materials.

Refer to the table below to understand the various standardized data sharing policy categories:


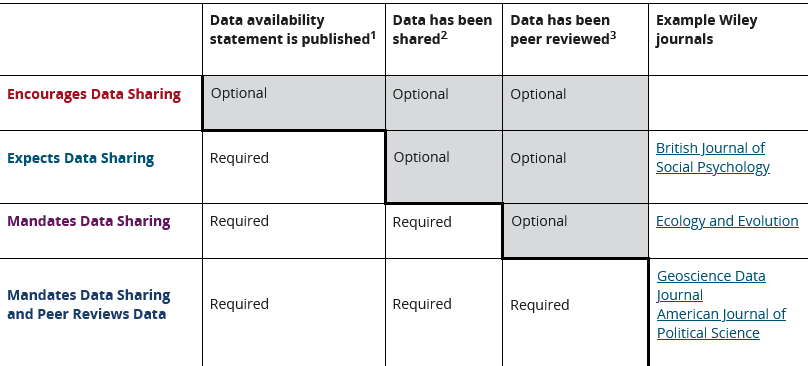


^1^ A data availability statement confirms the presence or absence of shared data.

^2^ Links to data in data availability statements are checked to ensure they link to the data that the authors intended. If data have been shared in a data repository, the data availability statement includes a permanent link to the data. Shared data is also cited.

^3^ Quality and/or replicability of linked data are peer reviewed. Depending on the journal, this may be to peer review the quality of the data by ensuring that the results in the paper and the data in the repository align (for example, sample sizes and variables match), or it may be to peer review the replicability of the data to ensure that the claims presented in the journal article are valid and can be reproduced.

### Mandates Data Sharing

The journal requires, as a condition for publication, that the data supporting the results in the paper will be archived in an appropriate public repository. Authors are required to provide a data availability statement, including a link to the repository they have used, and to cite the data they have shared. Whenever possible the scripts and other artefacts used to generate the analyses presented in the paper should also be publicly archived. Exceptions may be granted at the discretion of the editor, for example, if sharing data compromises privacy of human data, ethical standards or legal requirements. If authors are unable to share data (for example, if sharing data compromises ethical standards or legal requirements) then authors are not required to share it and must describe restrictions in their data availability statement.

See the Standard Templates for Author Use section below to select an appropriate data availability statement for your dataset.

### Standard Templates for Author Use

Below is a list of standard templates for the text that will appear in the "Data Availability Statement" portion of your article.. These statements adhere to guidelines set forth to comply with journals that have an "Expects Data" or "Mandates Data" policy.


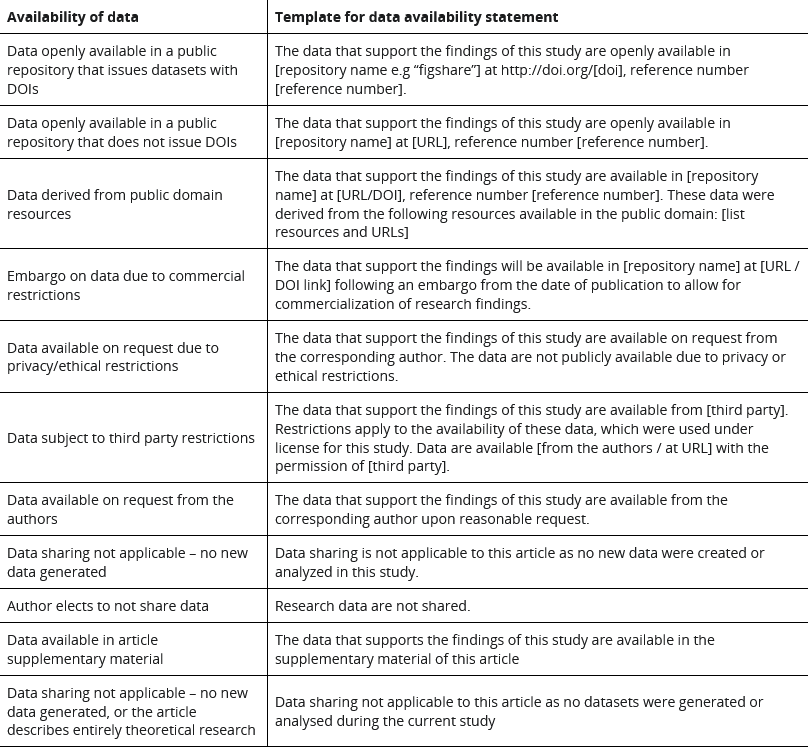


When data is available and linked, authors will need to provide a citation of the data in their reference list.

**Data citation:**

[dataset]Authors; Year; Dataset title; Data repository or archive; Version (if any); Persistent identifier (e.g. DOI)

The term [Dataset] will be removed before publication.

### How to choose an appropriate data repository

See below for Wiley’s recommended methods of choosing an appropriate data repository for your research:

- Visit our Author Compliance Tool (<https://authorservices.wiley.com/author-resources/Journal-Authors/open-access/author-compliance-tool.html> ) to check the data sharing policy of your chosen journal and/or funder before submitting your work
- Visit re3data.org or fairsharing.org to help identify registered and certified data repositories relevant to your subject area

# 3. Ecological Monographs

<https://www.esa.org/publications/data-policy>

## Open Research Policy

### OVERVIEW

ESA has adopted a society-wide Open Research Policy for its publications to further support scientific exploration and preservation, allow a full assessment of published research, and streamline policies across our family of journals. An open research policy provides full transparency for scientific data and code, facilitates replication and synthesis, and aligns ESA journals with current standards. As of 1 February 2021, all new manuscript submissions to ESA journals must abide by the following policy.

As a condition for publication in ESA journals, all underlying data and novel statistical code pertinent to the results presented in the publication must be made available in a permanent, publicly accessible data archive or repository upon acceptance of a manuscript, with rare exceptions (see the “Details” section for more information). Archived data and novel statistical code should be sufficiently complete to allow replication of tables, graphs, and statistical analyses reported in the original publication, and perform new or meta-analyses. As such, the desire of authors to control additional research with these data and/or code shall not be grounds for withholding material.

Thus, for the purpose of this policy, the following underlying material is required:

- Raw data and metadata used to generate tables, figures, plots, videos/animations
- Novel code or computer software utilized to generate results or analyses
- All methods or protocols utilized to generate the data, both existing (including references) and new methods/protocols
- Derived data products

Please see the “Details” section for definitions of each bulleted item above.

For submissions made prior to 1 February 2021 and revisions and/or accepted versions of those submissions:

- Data archiving remains optional for Ecosphere, Frontiers in Ecology and the Environment, and the ESA Bulletin.
- For Ecology, data and code archiving remains optional with the exception of “Data Papers” (data required) and “Statistical Reports” (code required).
- For Ecological Applications and Ecological Monographs, data archiving remains required per the previous policies for these journals (Ecological Applications policy for submissions prior to 1-Feb-2021; Ecological Monographs policy for submissions prior to 1-Feb-2021).

### DETAILS

#### All other ESA Journals Submissions

Raw data, metadata, and derived data products must be provided in an appropriate external archive if a manuscript is accepted. At the submission stage for most ESA manuscript types and journals (see the exceptions above), data should not be uploaded alongside your submission in the file list. Authors are not required to provide data at the submission stage, but it is strongly encouraged that authors deposit data in an external repository prior to manuscript submission to allow Subject-matter Editors and reviewers access. Many repositories now offer a private-for-peer-review option and we strongly encourage authors to use this approach.

Novel code must be supplied as private-for-peer review in an external repository during the review process. At the submission stage, code should not be uploaded with your submission in the file list.

#### Supporting Information Restrictions

In addition to our requirements for the presentation and archival of data and novel code, restrictions on the inclusion and formatting of specific pieces of supporting information are now in effect. The following items can only be made available as part of our Open Research Policy and cannot be included with a submission in the file list:

1) Spreadsheets (.xlsx, .csv, etc.)

2) Large tables

If the above items are included with your submission, your manuscript will be returned to you to ensure it follows our Open Research policy guidelines. The “Appendix” and “Data” designations should be considered a simple naming convention used to identify which material will be presented in PDF format (appendices) or spreadsheet format (data). For a definition of a “large table” please see “How do I know if my table is allowed as supporting information?” in our FAQ section.

#### After Acceptance

Following manuscript acceptance, complete data and novel code must be registered in a repository to be made available at the time of publication. ESA Publications staff will verify data and code archiving is complete before releasing files to the publisher. By depositing data and code prior to publication of a manuscript, a permanent link and formal citation can be included in the published paper.

#### Exceptions

Exceptions to this policy are granted only in rare cases as follows and must be fully disclosed by the author at the time of submission.

- Sensitive and confidential information should be redacted as required, including but not limited to precise locations of sampling on private lands, indigenous territory, or sacred sites; locality data for rare, threatened, or endangered species; identity of human subjects.
- In cases in which authors are not the legal data owner and cannot release the data (for example, commercial sources for fish landings), the author should provide sufficient query information so another researcher could seek to obtain the same data, and such limitations must be disclosed at the time of manuscript submission.
- All data on human subjects need to be anonymized to protect the identification of study participants; data from human subjects must be accompanied by an approval statement of the research project by the author’s institutional ethics review committee.

#### Definitions

Raw data and metadata: Raw data are not summarized data in the form of tables of means and standard deviations. Raw data are those used to perform statistical analyses and enable reuse for new or meta-analyses. Data should be provided in non-proprietary, machine-readable formats (e.g., “.csv”). Archived data sets must be accompanied by clear and precise metadata that allow others to readily use files, describing each column of a data set (e.g., locations; elements of experimental design; experimental units; measured variables, methods to measure each variable and units; species abbreviations; etc.).

“Novel” Code: To ensure appropriate code is presented to our readers, any novel code associated with the paper must be provided with the initial manuscript submission for peer review and editorial approval. Novel code is any code that cannot be directly cited and falls under two categories:

- A function or package you have created yourself
- A function or package you have edited to the extent that you can’t simply cite an existing function or package

We require a version of record (the exact version described in the paper that allows a reader to replicate the results presented) provided in the native format of the code file.

This material must be provided in an external repository during the peer review process. We strongly encourage using a repository with a private-for-peer review option, but a public repository posting is also acceptable. If the manuscript is accepted, we require publication of the code in an external repository that permits future updates with versioning control. Novel code in an outside repository must also be properly cited and referenced in the final publication. Code that is not novel (e.g., from a standard statistical package or publicly available model) must be properly cited and referenced.

If your code is not novel, please state this clearly in your Open Research statement. If your statement is ambiguous, the journal’s editorial staff will contact you for further details.

Methods or protocols: Use of known methodology/protocols should be cited appropriately with complete references. Detailed information describing new methodology should be presented in the main text (such as within a “Methods” section) or within an Appendix to be published alongside the manuscript as Supporting Information.

Derived data products: This constitutes data which are collected from database source(s) and collated and/or processed specific to the current research. Complete data source documentation and metadata are required, along with formal citation references. Details regarding data processing steps and assumptions should be clearly defined.

### CHOOSING A REPOSITORY

Data and code must be deposited in a permanent, trusted repository. These guidelines must be followed for both final storage and any material provided as private-for-peer review.

Personal author pages on institutional websites (such as a lab or project page) do not suffice, nor do generic cloud-storage services (such as Google Drive, Box, Sharepoint, Nimbus, or Dropbox), even if these services are licensed through a university or institution.

ESA maintains an extensive list of approved repositories; we encourage you to peruse the General Tips below and contact us with any general questions.

Authors will be responsible for any fees charged by external repositories to comply with the Open Research requirement.

Please note that the repository you select must present the material in English or offer a translation to English.

#### General Tips:

- To guarantee reviewer anonymity, repositories that are used for “private-for-peer review” sharing cannot require a reviewer to log in. Please see our FAQ section (“What are the requirements for “private-for-peer review” storage?”) for more information.
- A trusted repository will assign a DOI or other permanent identifier to the material and guarantees the material to be available in perpetuity.
- When possible, choose a repository that specializes in your scientific field to promote interoperability and reuse.
- Use of a repository run by the institution/affiliation/government agency of the author(s) is encouraged; this staff can be an excellent support system and help authors comply with institute and/or funder requirements.
- The Dryad data repository provides a flexible platform for a wide variety of digital data.
- A no-cost general repository option such as Figshare can be utilized.
- Other examples of permanent repositories include GenBank for DNA sequences, ORNL-DAAC for biogeochemical data, Knowledge Network for Biocomplexity, the NSF Arctic Data Center, and the Environmental Data Initiative (EDI).
- A Zenodo DOI must be obtained for GitHub material to ensure permanency and versioning. Instructions for obtaining a Zenodo DOI can be found here (<https://docs.github.com/en/repositories/archiving-a-github-repository/referencing-and-citing-content> ).

### MECHANICS AND EXAMPLES

#### Online submission form in the ScholarOne system:

At the new submission and revision stages, please consult the numbered list below. After determining the options that apply to you, please do the following: i) Note which options apply to you and disclose the current/future availability of data and code, affirming as many as needed in the Comment Box of the “Open Research” section of the ScholarOne online submission form, AND ii) Add an Open Research statement on the manuscript’s title page that includes any extra requirements for your choice(s) for disclosing the availability of data and code. Submission requirements for each choice are shown in [**bold**].

1. No data were collected for this study (i.e., theoretical, review, opinion, editorial papers).

2. Data are already published and publicly available, with those publications properly cited in this submission. **[If choosing this option, include complete citations for the publicly available data sets you used for your manuscript. You must also include complete citation entries in the References section when possible and cite as appropriate in the main text. If the material was retrieved from an external database that does not use permanent identifiers for datasets, you must provide the necessary query details another qualified researcher would need to obtain the same data you accessed from the external database. Please note: This option is used only for material that is currently publicly available. Do not use this option to refer to the link where your data will eventually be made available if the manuscript is accepted.]**

3. Data are provided as private-for-peer review (shared privately or publicly on a repository). **[If choosing this option, in the Open Research statement you must provide an active link to where the material is currently stored and state the intended repository where data will be permanently archived if the paper is accepted for publication. Data files can NOT be uploaded with your manuscript in the system.]**

4. Data are not yet provided. **[If choosing this option, you must disclose why in the Open Research statement, affirm data will be permanently archived if the paper is accepted for publication, and note the intended repository. Stating data will only be provided upon acceptance of the manuscript is permissible but be advised the Subject-matter Editor or reviewers may request to see this data during review if deemed necessary.]**

5. Data are sensitive and cannot be provided publicly (rare; examples include sensitive species data and human subject data). **[If choosing this option, in the Open Research statement you must fully disclose what data are private and provide complete query and contact details so another qualified researcher can obtain the data. You must provide what data sets you can, with the sensitive data points redacted as necessary. This anonymized data must be provided following the standard archiving guidelines of our Open Research policy. ]**

6. This submission uses novel code, which is provided, per our requirements, in an external repository to be evaluated during the peer review process. **[Novel code files MUST be provided as private-for-peer review in an external archive. A repository with a private-for-peer review option is recommended (such as Dryad or Figshare), but a public repository such as GitHub is acceptable. If choosing this option, your Open Research statement must include the link to the private-for-peer review storage location and state the intended repository where the code will also be permanently archived if the paper is accepted for publication. If your code is not novel, please state this clearly in your Open Research statement.]**

#### Manuscript file:

The manuscript file should include an Open Research statement on the manuscript’s title page that fully describes where readers can access all underlying data and/or code. Statements to the effect of “material are available from the author(s)” are not permissible. Examples of Open Research statements for accepted papers are provided below.

At the submission stage, please consult the numbered list above and include a statement that meets one of these requirements.

For final submissions of accepted manuscripts, this statement must convey complete data archiving details, including data source citations and formal literature citation entries. Note any access restrictions.

#### Examples of text for the “Open Research” statement

**For data and/or code that are currently available in a repository as private-for-peer review:**

Data and/or code are provided as private-for-peer review via the following link: [Link to external storage location]

[Include an active link to the current location where data and/or code can be retrieved]

**For data that are not yet provided, but will be archived upon acceptance:**

Data are not yet provided [Include the reason that data are not yet provided]. Upon acceptance data will be archived in [Include the name of the external repository you will be using].

**For previously published data currently stored in a repository by authors of this research paper:**

Data (Smith et al. 2019) are available from Dryad: [Dryad data set DOI]

[Include complete Smith et al. 2019 data set entry in the Literature Cited and cite as appropriate in the main text. Please note: This option is used only for material that is currently publicly available. Do not use this option to refer to the link where your data will eventually be made available if the manuscript is accepted.]

**For data previously released and published by individuals other than authors of this research paper:**

Data sets utilized for this research are as follows: [citation(s) with DOI/URL data set links]

[Include complete citation entries in the Literature Cited when possible and cite as appropriate in the main text]

**For data retrieved from a public database:**

[Description of specific dataset retrieved] was retrieved from [name of database, including hyperlink]. Query details for retrieving the relevant data are as follows: [provide the necessary query details another qualified researcher would need to obtain the same data].

[Query details should give specific information to direct a reader to download the same data sets you used for your manuscript and should be tailored to the database. Examples of query details include, directions to search pages, search terms used in web forms, the specific names of data sets you used from an available collection, etc. You should not simply state what data in your manuscript came from the database; you should describe how to retrieve it from the database.

If the query details are especially lengthy or complex, you can instead include the specific query details in an appendix and use the following format for the final sentence “Query details are described in [the appendix location where query details are provided]”. This should point to a specific point of your appendix (such as a table or labeled section) where a reader can find the query details.]

**For most theoretical, review, opinion, or editorial papers, and Bulletin Photo Gallery submissions:**

Empirical data were not used for this research.

**(Rare) For data not publicly available, but available to researchers with appropriate credentials:**

Data are not publicly available due to [fill in reasons, such as sensitive species data]. Data can be obtained from [provide complete query and contact details, with license and access restrictions, if any]. Upon acceptance, anonymized data will be archived in [Include the name of the external repository you will be using].

**(Rare) For data that are restricted by commercial, industry, patent, government policies, regulations, and/or laws:**

Data supporting this research are available from [third party source], with [restrictions, including non-disclosure agreements, licensing, other agreements], and are not accessible to the public. [Provide full query and contact process details for other researchers to gain access.]

NOTE: If your data are in either of the “Rare” statement categories, the Subject-matter Editor will be asked to determine if the statement provides the detail necessary to meet ESA guidelines.

### Frequently Asked Questions

This section is a living document, and we intend to expand the list. Additional questions can be submitted to esajournals@esa.org

**Q: What are the advantages of posting my data and code on a public repository?**

A: Advantages of using a permanent repository include:

- Visibility: Making your data/code available online (and linking it to the journal publication) provides a new pathway for others to learn about your work.
- Citability: All data/code you deposit will receive a persistent, resolvable identifier that can be used in a citation.
- Workload reduction: If you receive individual requests for data/code, you can simply direct them to files in the archive.
- Quality control: storage of data in a permanent public repository encourages complete transparency and discourages the falsification of data and other fraudulent publication practices in ESA journals.
- Preservation: Your data/code files will be permanently and safely archived in perpetuity.
- Impact: You will garner citations for both the research paper and data/code product through the reuse of your data/code.
- Potential for greater collaboration: Other scientists searching for data to perform meta-analyses or develop new hypotheses may involve you as a collaborator and author.

**Q: Do I need to archive my data prior to manuscript submission?**

A: Only for “Data Papers” and “Statistical Reports” in Ecology. For other manuscript types and journals, it is not required at submission but is strongly encouraged. If you are depositing data in a repository at the manuscript submission stage, we suggest keeping it “private for peer review” and this data deposit should not specify the journal since the paper is not formally accepted. Except for “Data Papers” and “Statistical Reports” in Ecology, at the submission stage we only require confirmation of understanding of the policy and mention of the intended repository for verification purposes.

**Q: Do I need to provide my novel code at the time of manuscript submission?**

A: Yes, novel code is required in order to be evaluated. Just like the main manuscript and other files, this material is provided to reviewers as part of the reviewing package. Novel code must be made accessible via an external archive during all stages of the review process. A repository with a private-for-peer review option is recommended (such as Dryad or Figshare), but a public repository such as GitHub is acceptable.

**Q: How should I prepare my code?**

A: Best practices guidelines advise authors to use a scriptable statistical environment, generating statistical code that is sufficiently complete to allow replication of tables, graphs, and statistical analyses reported in the original publication and perform new or meta-analyses. Every number, every P value, and every graph requires formal archiving of the original script that generated it. In cases in which published results are challenged or questioned, these scripts will be used to reconstruct the published analyses, which is the critical first step towards resolving the dispute.

**Q: What are the requirements for sharing novel code (required) and data (not required) during the review process?**

A: Authors are not required to provide data as during the review process at initial submission but it is strongly encouraged. Novel code, however, must be provided during the review process.

If an author provides material during the review process, the repository must guarantee reviewer anonymity. A repository cannot require a reviewer to log in to access the material. Personal author pages on institutional websites (such as a lab or project page) also do not suffice, nor do generic cloud-storage services (such as Google Drive, Box, Sharepoint, Nimbus, or Dropbox), even if these services are licensed through a university or institution.

The best practice is to use a repository that provides a direct link to the “private-for-peer review” material with no log-in required, such as Figshare or Dryad. A public-facing repository such as GitHub will also be acceptable.

**Q: What do you mean by “private-for-peer review”?**

A: “Private-for-peer review” refers to the process of storing material temporarily as private during the review process, with access provided by a direct anonymous link to the material. For material that is provided during the review, private-for-peer review options are recommended (such as Dryad or Figshare), but a public repository where material can be viewed anonymously, but it is not stored privately (such as GitHub) is acceptable. We do not allow generic cloud-storage services for private-for-peer review or for final archival of material, even if these services are licensed through a university or institution.

**Q: What are the requirements for “private-for-peer review” storage?**

A: Authors are not required to provide data as “private-for-peer review” at initial submission but it is strongly encouraged. Novel code, however, must be provided as private-for-peer review.

If an author provides material as “private-for-peer review”, the repository must guarantee reviewer anonymity. A repository cannot require a reviewer to log in to access the material as “private-for-peer review”. Personal author pages on institutional websites (such as a lab or project page) also do not suffice, nor do generic cloud-storage services (such as Google Drive, Box, Sharepoint, Nimbus, or Dropbox), even if these services are licensed through a university or institution.

The best practice is to use a repository that provides a direct link to the “private-for-peer review” material with no log-in required, such as Figshare or Dryad. A public-facing repository such as GitHub be acceptable.

**Q: What happens if I have archived my data and my paper is not accepted by the journal?**

A: Your data on the repository remains there and can be linked to a new submission at a different journal. Your data deposited on a repository should not specify a related journal publication until the paper is formally accepted.

**Q: Can I provide my data or code after my accepted manuscript is released online?**

A: No, data and code links must be verified and included in the final file sent to the publisher. Authors will need to archive data/code as part of the final submission process. Manuscript files will not be sent to the publisher for typesetting until this requirement is met.

**Q: Can I embargo the data or code associated with my accepted manuscript in order to release the data or code at a later time?**

A: No, we do not permit any kind of data or code embargo following manuscript acceptance. Related data and code must be publicly available before the final manuscript is sent to the publisher for typesetting. The desire of authors to control additional research with these data and/or code shall not be grounds for withholding material.

**Q: What should I name my data or code package in the external repository?**

A: To avoid confusion and possible errors, data and code packages should not be named the exact same name as your manuscript title. Use a descriptive name for data and code packages which meets the guidelines of the repository. A suggested format is “Data from: [manuscript title]” or “Code for: [manuscript title]”. Do include a reference to your related ESA journal publication in data and code deposits.

Individual files in your data or code deposit should be given unique and descriptive file names that can be referred to when necessary (for example: “Ungulate_geospatial_data.xlsx”). Please do not use the format “Data S#”, “Appendix S#”, “Table S#”, or any similar “Item S#” name for the material being placed on a data or code repository, as this naming convention is used by the publisher for supporting information hosted on the journal platform and can result in confusion during file processing and for readers at all stages.

**Q: What repository should I use if I have a very large dataset?**

A: In previous cases, authors with very large datasets in the 100s of GB range have successfully used Figshare or Knowledge Network for Biocomplexity to meet the requirements of our Open Research policy.

**Q: How do I know if my table is allowed in an appendix as supporting information?**

A: Appendix tables cannot be provided as spreadsheet files. If your table requires a spreadsheet format, it must be provided under the Open Research policy. Appendix tables will be published in PDF format and should be presented in the clearest and most readable format possible, with font sizing at 8-pt or larger. Appendix tables can be no longer than two pages. If an appendix table is too large, staff may require you remove it and provide it as data under the Open Research policy for ease of re-use. Exception: “Data Papers” in Ecology should provide data in spreadsheet format.

**Q: Will ESA help me craft my Open Research statement?**

A: Please make every effort to review and follow the statement examples provided in the “Mechanics and Examples” section. After you have submitted your manuscript, your statement will be checked by the journal’s editorial staff and they will request clarifications if necessary. Specific questions can be directed to the journal’s editorial staff .

**Q: The repository requires the journal publication DOI before releasing the data. How can I obtain the DOI for my ESA journal paper to link to it?**

A: The publisher will assign the DOI shortly after their receipt of the files, and most data repositories can amend the data release to include this detail when requested to do so. Contact the journal’s editorial staff if your paper is accepted and your repository is not able to accommodate this.

**Q: ESA will link the paper to the data set, but how can I link the data set to the paper?**

A: Please work with your repository to link the data set back to the journal release. This requires some finesse because the journal article will not yet be released.

**Q: I would like to deposit data in Dryad. Does your journal provide manuscript information to them?**

A: Yes! Please let us know you will be using Dryad when uploading your manuscript submission, and we can simplify your data submission process with Dryad. Detailed Dryad submission instructions will be provided at manuscript acceptance.

**Q: Are there fees associated with depositing data?**

A: Authors are responsible for fees associated with data deposits. Authors of Ecological Monographs contributions that choose to deposit data in Dryad will be assessed a reduced fee. Repository fees typically range from $0 to $120 for most data sets, with structured, incremental fee increases for very large deposits (these are generally structured by ranges of gigabytes). Sites like Figshare are a no-cost option, as are many university library repositories.

**Q: When referring to the published paper and/or data set, should I cite the paper, the data set, or both?**

A: Both items are considered research output, and both should be cited as appropriate.

**Q: What precautions should be taken with sensitive human subject data?**

A: If you work with human subjects, be sure to follow all relevant guidelines for the protection of personally identifying information (PII) as specified in your Institutional Review Board (IRB) application and approval. Data need to be sufficiently anonymous so that identity cannot be surmised from the data set.

**Q: What additional resources are available?**

A: We encourage authors to explore the FAIR principles and Powers and Hampton (2019) in Ecological Applications (Open Access: https://doi.org/10.1002/eap.1822). Authors, editors, reviewers, and readers are encouraged to reach out to the journal’s editorial staff with any questions for manuscript preparation or regarding previously published materials.

# 4. Ecology and Evolution

<https://onlinelibrary.wiley.com/page/journal/20457758/homepage/forauthors.html>

## Policy on Data Archiving

Ecology and Evolution requires authors to (a) provide all data, metadata and code on submission for review by editors and referees and (b) adhere to our minimum standards in data and code archiving set out here.

In brief, authors are required to format their open data to these standards, archive them in a publicly accessible repository, and clearly state in their submission where they are deposited. Upon acceptance, data must be formally archived and the Data Accessibility Statement completed with links to all open data from the manuscript. Manuscripts submitted without data will not be passed through to an editor. If you provide your data as supplementary files, please state this in your Data Accessibility Statement.

If authors choose to use the Dryad data repository, Ecology and Evolution will pay the archiving charges on their behalf if their paper is published in the journal. If you use Dryad, please include the “Private for Peer Review” link in your Data Availability Statement to make your data accessible to reviewers.

Acceptable reasons for exemptions are laid out in our minimum standards. If you believe you have a case for an exemption, please clearly state so in your cover letter.

## Open Research Badges

In partnership with the non-profit Center for Open Science (COS), Ecology and Evolution offers all submitting authors access to the following three Open Research Badges— Open Materials, Open Data, and Preregistered Research Designs. We also award all qualifying authors Open Research Badges recognizing their contributions to the Open Research movement. The Open Research practices and associated award badges, as implemented by the Center for Open Science and supported by Ecology and Evolution, are the following:

The Open Materials Badge recognizes researchers who share their research instruments and materials in a publicly-accessible format, providing sufficient information for researchers to reproduce procedures and analyses of published research studies.

The Open Data Badge recognizes researchers who make their data publicly available, providing sufficient description of the data to allow researchers to reproduce research findings of published research studies.

The Preregistered Badge recognizes researchers who preregister their research plans (research design and data analysis plan) prior to engaging in research and who closely follow the preregistered design and data analysis plan in reporting their research findings. The criteria for earning this badge thus include a date-stamped registration of a study plan in such venues as the Open Science Framework (https://osf.io) or Clinical Trials (https://clinicaltrials.gov) and a close correspondence between the preregistered and the implemented data collection and analysis plans.

Authors will have an opportunity at the time of manuscript submission and at the time of acceptance to inform themselves of this initiative and to determine whether they wish to participate. Applying and qualifying for Open Research Badges is not a requirement for publishing with Ecology and Evolution, but these badges are further incentive for authors to participate in the Open Research movement and thus to increase the visibility and transparency of their research.

More information about the Open Research Badges is available from the Open Science Framework wiki (<https://osf.io/tvyxz/wiki/home/> ).

## Data Sharing and Data Availability

This journal mandates data and peer reviews data sharing. Review Wiley’s Data Sharing policy (<https://authorservices.wiley.com/author-resources/Journal-Authors/open-access/data-sharing-citation/data-sharing-policy.html> ) where you will be able to see and select the data availability statement that is right for your submission.

## Wiley’s Data Sharing Policies

Wiley is committed to a more open research landscape, facilitating faster and more effective research discovery by enabling reproducibility and verification of data, methodology and reporting standards. We encourage authors of articles published in our journals to share their research data including, but not limited to: raw data, processed data, software, algorithms, protocols, methods, materials.

Refer to the table below to understand the various standardized data sharing policy categories:


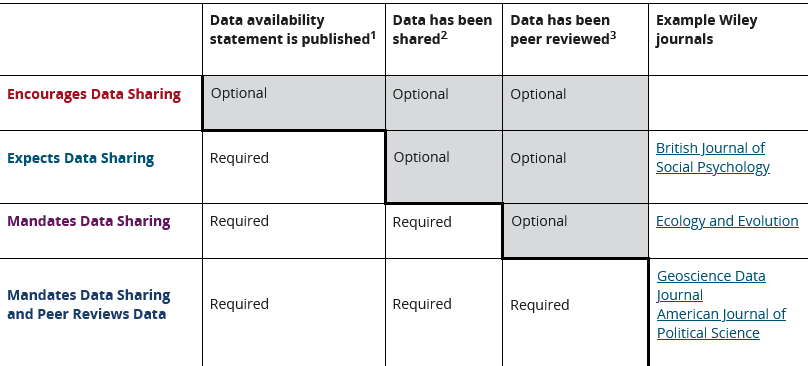


^1^ A data availability statement confirms the presence or absence of shared data.

^2^ Links to data in data availability statements are checked to ensure they link to the data that the authors intended. If data have been shared in a data repository, the data availability statement includes a permanent link to the data. Shared data is also cited.

^3^ Quality and/or replicability of linked data are peer reviewed. Depending on the journal, this may be to peer review the quality of the data by ensuring that the results in the paper and the data in the repository align (for example, sample sizes and variables match), or it may be to peer review the replicability of the data to ensure that the claims presented in the journal article are valid and can be reproduced.

### Mandates Data Sharing

The journal requires, as a condition for publication, that the data supporting the results in the paper will be archived in an appropriate public repository. Authors are required to provide a data availability statement, including a link to the repository they have used, and to cite the data they have shared. Whenever possible the scripts and other artefacts used to generate the analyses presented in the paper should also be publicly archived. Exceptions may be granted at the discretion of the editor, for example, if sharing data compromises privacy of human data, ethical standards or legal requirements. If authors are unable to share data (for example, if sharing data compromises ethical standards or legal requirements) then authors are not required to share it and must describe restrictions in their data availability statement.

See the Standard Templates for Author Use section below to select an appropriate data availability statement for your dataset.

### Standard Templates for Author Use

Below is a list of standard templates for the text that will appear in the "Data Availability Statement" portion of your article.. These statements adhere to guidelines set forth to comply with journals that have an "Expects Data" or "Mandates Data" policy.


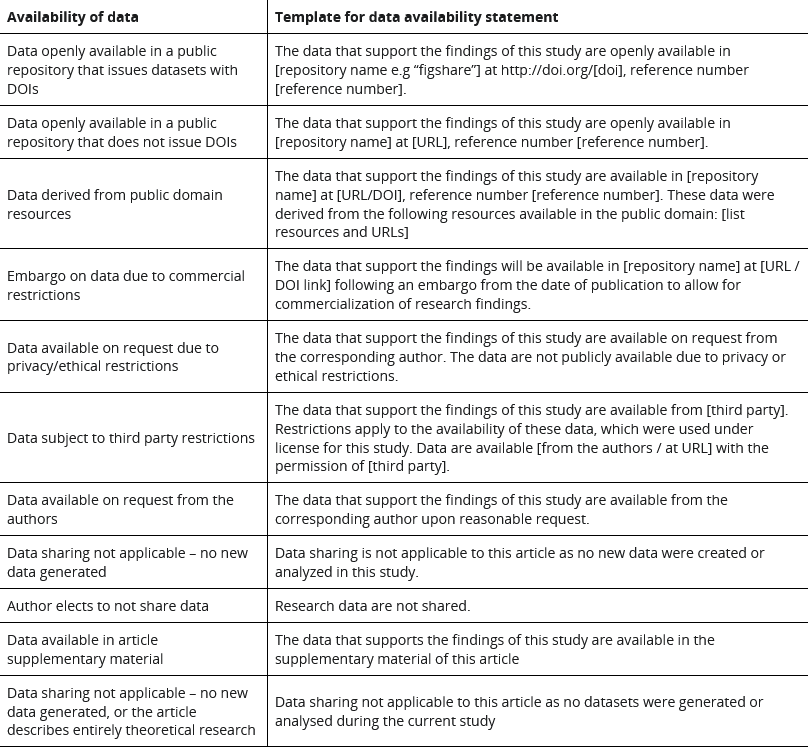


When data is available and linked, authors will need to provide a citation of the data in their reference list.

**Data citation:**

[dataset]Authors; Year; Dataset title; Data repository or archive; Version (if any); Persistent identifier (e.g. DOI)

The term [Dataset] will be removed before publication.

### How to choose an appropriate data repository

See below for Wiley’s recommended methods of choosing an appropriate data repository for your research:

- Visit our Author Compliance Tool (<https://authorservices.wiley.com/author-resources/Journal-Authors/open-access/author-compliance-tool.html> ) to check the data sharing policy of your chosen journal and/or funder before submitting your work
- Visit re3data.org or fairsharing.org to help identify registered and certified data repositories relevant to your subject area

## Privacy/Data protection

By submitting a manuscript to or reviewing for this publication, your name, email address, affiliation, and other contact details the publication might require, will be used for the regular operations of the publication, including, when necessary, sharing with the publisher (Wiley) and partners for production and publication. The publication and the publisher recognize the importance of protecting the personal information collected from users in the operation of these services, and have practices in place to ensure that steps are taken to maintain the security, integrity, and privacy of the personal data collected and processed. You can learn more at: https://authorservices.wiley.com/statements/data-protection-policy.html

# 5. Ecology Letters

<https://onlinelibrary.wiley.com/page/journal/14610248/homepage/forauthors.html#data-availability>

## 19. DATA AND CODE AVAILABILITY

Data and code are important products of scientific enterprise, and they must be preserved and remain accessible in future decades.

For manuscripts that depend on new or existing data, and/or on code written by the authors, Ecology Letters requires that this material be supplied and accessible to editors and reviewers at the time of submission. This means that prior to submission, you will need to prepare your raw data (or the subset of existing data that was used), metadata, code and derived data products, archive this material in an external repository that subject editors and reviewers can access, and include a clear README file that will ensure accessibility and readability. This will allow us to properly assess your work and confirm that the archive is complete and usable. See below for more details on the requirements.

Ecology Letters requires that the raw data (or subset of existing data) used to generate the results in the paper are archived in one of the following public repositories: Dryad, Figshare, Hal, Zenodo, OSF, US federal agencies repositories, or Environmental Data Initiative (EDI). Data should not be uploaded alongside your submission as a supporting document. In order to ensure data can be reusable, it is necessary to upload (1) the data files that were analysed to produce the statistics and figures reported in the paper, (2) a metadata file explaining what the data are in each column and what rows represent. The metadata should record the units of measurement and a verbal description in English of the data included in each column. Dryad also publishes a list of best practices (<https://datadryad.org/stash/best_practices> ) that Ecology Letters endorses.

Computer code used to produce the figures and conduct analyses or simulations must also be archived in a public repository (e.g., Zenodo, Figshare). Code should not be uploaded with your submission as a supporting document. All code must be annotated so readers can understand what each segment or function does. If figures have been generated in a program such as Excel, the Excel file including the figure and the data must be archived. Further guidance is available at <https://authorservices.wiley.com/author-resources/Journal-Authors/open-access/data-sharing-citation/index.html>.

If the raw data used for a study are already deposited online in a different repository (excluding business-owned and single-university repositories) with a separate DOI, this can be cited. However, often data sources used from an already published dataset are quite different than the original, using different subsets of the data and manipulating it in different ways. In such cases, we request that authors of Ecology Letters papers follow best practices for open science and provide code (preferably in R) that is able to access the cited DOI, retrieve the raw data used for the study, and manipulate it into the form used for analyses. This data accessing code must be combined with the code used for analyses, and deposited in the same repository.

DNA sequences published in Ecology Letters must be deposited in the EMBL/GenBank/DDJB Nucleotide Sequence Databases. An accession number for each sequence must be included in the manuscript.

Exceptions may be granted at the discretion of the Editor-in-Chief for sensitive information such as human subject data or the location of endangered species. An embargo of up to one year is permitted. Requests for longer periods will be rejected. At the time of submission, authors must use the private-for-peer-review option provided by data repositories.

Authors are required to complete a data accessibility statement for all papers, confirming that each of these actions has been undertaken. The statement, as well as data and code DOI, must be provided at initial submission. Editors will screen some of the data submitted. If at a later date it is found that authors of published papers have not adhered to these strict guidelines, the paper will be retracted by the editors and a statement made in the journal justifying this action.

# 6. Evolution Letters

<https://academic.oup.com/evlett/pages/general-instructions#Availability_of_data_and_materials>

## Availability of data and materials

Evolution Letters requires all authors, where ethically possible, to publicly release all data and software code underlying any published paper as a condition of publication. Authors are required to include a data availability statement in their article. When data and software underlying the research article are available in an online source, authors should include a full citation in their reference list. For details of the minimum information to be included in data and software citations see the OUP guidance on citing research data and software (<https://academic.oup.com/pages/open-research/research-data#Citing%20research%20data%20and%20software> ).

Wherever possible, data should be presented in the main manuscript, included as supplementary material, or deposited in a public repository. Information on general repositories for all data types, and a list of recommended repositories by subject area, is available on the OUP Research data page (<https://academic.oup.com/pages/open-research/research-data#data2> ).

Authors may request an exception to the policy due to legal, privacy, ethical, or other limitations or restrictions. Exceptions will be made at the discretion of the Journal. Please notify the editorial office when submitting your manuscript if you wish to request an exception. If an exception is granted, a data and code availability statement must still be included in your paper specifying what cannot be shared and explaining why.

### Data availability statement

The inclusion of a data availability statement is a requirement for articles published in Evolution Letters. Data availability statements provide a standardized format for readers to understand the availability of original and third-party data and code underlying the research results described in the article. The statement should describe and provide means of access, where possible, by linking to the data or providing the required unique identifier.

The data availability statement should be included in the end matter of your article under the heading “Data and code availability”.

More information and example Data availability statements can be found here (<https://academic.oup.com/pages/open-research/research-data> ).

### Data citation

Evolution Letters supports the Force 11 Data Citation Principles (<https://www.force11.org/group/joint-declaration-data-citation-principles-final> ) and requires that all publicly available datasets be fully referenced in the reference list with an accession number or unique identifier such as a digital object identifier (DOI). Data citations should include the minimum information recommended by DataCite (<http://www.datacite.org.s3-website-eu-west-1.amazonaws.com/cite-your-data.html> ):

[dataset]* Authors, Year, Title, Publisher (repository or archive name), Identifier

*The inclusion of the [dataset] tag at the beginning of the citation helps us to correctly identify and tag the citation. This tag will be removed from the citation published in the reference list.

### Analytic methods (code) and materials storage and documentation

The policy of Evolution Letters is to publish papers in which authors make their empirical raw data and analytic methods available to other researchers for the purposes of reproducing the results or replicating the procedure. Authors must specify where that material is available in their data availability statement. There are several generalist data repositories where you may wish to consider archiving your data. Most of these can also host R objects and R scripts.

- Dryad Digital Repository
- Zenodo (can be integrated with a Dryad dataset)
- Figshare
- GitHub
- Open Science Framework

For R packages (which can include R objects, scripts, and documentation), repositories such as CRAN, BioConductor, and GitHub are often used.

Note, as part of our ongoing commitment to the public archiving of scientific data, Evolution Letters has established a sponsorship agreement with Dryad and will sponsor the data archives of accepted manuscripts in Dryad at no cost to the authors.

### Meta-analyses and systematic reviews – PRISMA and citations

We strongly encourage authors of meta-analyses and systematic reviews to adhere to the Preferred Reporting Items for Systematic reviews and Meta-Analyses (PRISMA) 2020 framework. Authors should implement PRISMA guidelines in their manuscript and provide a completed PRISMA checklist as a supplementary file.

Authors should include only the citations referred to in the main manuscript in the manuscript reference list. Where the total number of referenced sources is ≤ 50, authors are encouraged to incorporate citations into the main text, preferably either in a main-text table (where the number is <20) or in an appendix table (where the total is 20–50). If there are more than 50 sources to be cited, they should be listed in a table in the supplementary materials. A complete reference list for all sources referred to in the supplementary materials must be provided in a supplementary materials file.

### Study and analysis transparency

The policy of Evolution Letters is to publish papers where authors follow standards for disclosing key aspects of the research design and data analysis. Authors are encouraged to review the Tools for Transparency in Ecology and Evolution (TTEE) (<https://osf.io/g65cb/wiki/home/> ) or the standards available for many research applications from the Equator network (<https://www.equator-network.org/> ), and use those that are relevant for the reported research applications.

## Oxford University Press (OUP) policy

<https://academic.oup.com/pages/open-research/research-data#data2>

## Research data

### Research data for journals

Oxford University Press (OUP) and the journals we publish enforce high standards of rigour in the materials we accept for publication. The availability of data, code and other materials underlying research publications offers greater transparency, better trust in the literature and better reproducibility. Reuse of data offers enormous potential for further scientific and economic development. Making available the data underlying research publications supports our educational and scholarly mission as a department of the University of Oxford.

OUP is a signatory to the Center for Open Science’s Transparency and Openness Promotion (TOP) Guidelines which served as a framework for our overall data policy approach. OUP also supports the Joint Declaration of Data Citation Principles and the recommendations of the FORCE11 Software Citation Implementation Group, as reflected in our data and software citation practices.

### Data availability policies

OUP encourages authors, where ethically feasible, to make the research data underlying their articles publicly available. Because the nature of research data varies widely between different disciplines, each individual journal has set a field-appropriate data availability policy based on one of the levels listed below. Please see the individual journal’s Instructions to Authors for further details on the data requirements and citation practices of that publication.

Level 1: The journal encourages all authors, where ethically possible, to publicly release all data underlying any published paper.

Level 2: The journal encourages all authors, where ethically possible, to publicly release all data underlying any published paper. Authors must include a Data Availability Statement in their published article.

Level 3: The journal requires all authors, where ethically possible, to publicly release all data underlying any published paper as a condition of publication. Authors must include a Data Availability Statement in their published article.

Level 4: The journal requires all authors, where ethically possible, to publicly release all data underlying any published paper as a condition of publication. The data must undergo peer review along with the manuscript as part of the acceptance process. Authors must include a Data Availability Statement in their published article.

### Data Availability Statements

Data Availability Statements provide a standardised format to describe the availability of data underlying the research results of the article. The statement may refer to original data generated in the course of the study or to third-party data analysed in the article. The statement should describe and provide means of access, where applicable, by linking to the data or providing the required unique identifier.

**Sample Data Availability Statements**

**
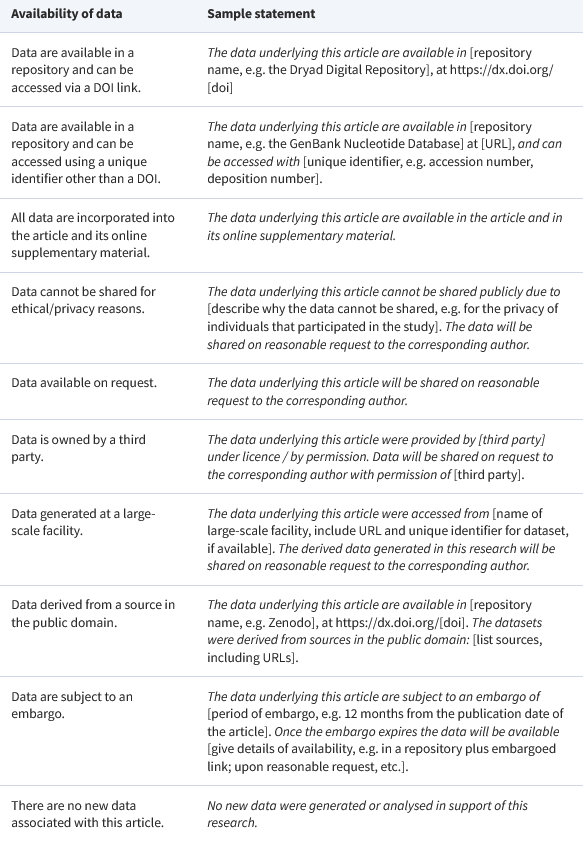
**

Please see the Instructions to Authors of your chosen journal for their specific requirements in relation to Data Availability Statements.

### Citing research data and software

OUP endorses the Joint Declaration of Data Citation Principles, and the recommendations of the FORCE11 Software Citation Implementation Group. Research data and software are legitimate, citable products of research for which citations should be given the same status as citations to publications.

In addition to references to data and software included in a data availability statement, when materials underlying the research article are available in an online source authors should include a full citation in their reference list. The citation should include a persistent link enabling readers to directly access the source, i.e. an online repository.

Citing data and software enables the authors to get credit for their contribution to the research article, and also increases the discoverability of the research data and software.

Data and software citations should include the following minimum information where available:

- Author(s) / Creator(s)
- Date
- Title / Software name
- Publication venue (e.g repository or archive name)
- Persistent identifier (e.g. DOI)

In addition, some journal reference styles may require the following information:

- Version number or date of access
- Bracketed description of material type (e.g. Dataset, Computer software)

*When citing software, if there is an article describing the software it is recommended to cite both the software and the article.

### Choosing where to archive your data

OUP encourages authors to share their data and other research materials to support the transparency and reproducibility of their research. Authors should consult the journal Instructions to Authors for recommendations/requirements on where to archive data. Many disciplines have specific requirements for depositing of data in recognised disciplinary repositories.

You may also refer to online resources such as FAIRsharing.org and re3data.org for lists of data repositories, including information on certification status and services offered.

Authors are highly encouraged to select a repository that issues DOIs as this helps to facilitate persistent linking to the dataset from the research article.

There are also a number of generalist data repositories that you may wish to consider when depositing your data:

- Code Ocean (for data and code)
- Dryad Digital Repository
- Figshare
- Harvard Dataverse
- Open Science Framework
- Zenodo
- Dryad Digital Repository

### Dryad Digital Repository

A number of Oxford University Press journals sponsor the deposition of data by their authors in the Dryad Digital Repository:

- Behavioral Ecology
- Evolution
- Evolution Letters
- JAMIA Open
- Journal of Experimental Botany
- Journal of the American Medical Informatics Association
- Journal of Heredity
- Ornithological Applications
- Ornithology
- Systematic Biology
- Toxicological Sciences

For more information see the journal Instructions to Authors.

# 7. Frontiers in Ecology and the Environment

<https://esajournals.onlinelibrary.wiley.com/hub/journal/15409309/resources/author-guidelines-FEE#open-research-policy>

## Open Research Policy

<https://www.esa.org/publications/data-policy>

All manuscript submissions must adhere to the ESA Open Research Policy. To do so, authors must include a statement of compliance at the time of submission. For authors whose data or code must be made available in an accepted publicly accessible archive, please note: we understand that authors often postpone undertaking the work necessary to provide archival access until after acceptance, but this works both ways: our peer review process relies on our AEs and reviewers being able to assess the data and/or code on which a manuscript is based. Therefore, access is needed prior to a decision being rendered on the submission.

### OVERVIEW

ESA has adopted a society-wide Open Research Policy for its publications to further support scientific exploration and preservation, allow a full assessment of published research, and streamline policies across our family of journals. An open research policy provides full transparency for scientific data and code, facilitates replication and synthesis, and aligns ESA journals with current standards. As of 1 February 2021, all new manuscript submissions to ESA journals must abide by the following policy.

As a condition for publication in ESA journals, all underlying data and novel statistical code pertinent to the results presented in the publication must be made available in a permanent, publicly accessible data archive or repository upon acceptance of a manuscript, with rare exceptions (see the “Details” section for more information). Archived data and novel statistical code should be sufficiently complete to allow replication of tables, graphs, and statistical analyses reported in the original publication, and perform new or meta-analyses. As such, the desire of authors to control additional research with these data and/or code shall not be grounds for withholding material.

Thus, for the purpose of this policy, the following underlying material is required:

- Raw data and metadata used to generate tables, figures, plots, videos/animations
- Novel code or computer software utilized to generate results or analyses
- All methods or protocols utilized to generate the data, both existing (including references) and new methods/protocols
- Derived data products

Please see the “Details” section for definitions of each bulleted item above.

For submissions made prior to 1 February 2021 and revisions and/or accepted versions of those submissions:

- Data archiving remains optional for Ecosphere, Frontiers in Ecology and the Environment, and the ESA Bulletin.
- For Ecology, data and code archiving remains optional with the exception of “Data Papers” (data required) and “Statistical Reports” (code required).
- For Ecological Applications and Ecological Monographs, data archiving remains required per the previous policies for these journals (Ecological Applications policy for submissions prior to 1-Feb-2021; Ecological Monographs policy for submissions prior to 1-Feb-2021).

### DETAILS

#### All other ESA Journals Submissions

Raw data, metadata, and derived data products must be provided in an appropriate external archive if a manuscript is accepted. At the submission stage for most ESA manuscript types and journals (see the exceptions above), data should not be uploaded alongside your submission in the file list. Authors are not required to provide data at the submission stage, but it is strongly encouraged that authors deposit data in an external repository prior to manuscript submission to allow Subject-matter Editors and reviewers access. Many repositories now offer a private-for-peer-review option and we strongly encourage authors to use this approach.

Novel code must be supplied as private-for-peer review in an external repository during the review process. At the submission stage, code should not be uploaded with your submission in the file list.

#### Supporting Information Restrictions

In addition to our requirements for the presentation and archival of data and novel code, restrictions on the inclusion and formatting of specific pieces of supporting information are now in effect. The following items can only be made available as part of our Open Research Policy and cannot be included with a submission in the file list:

1) Spreadsheets (.xlsx, .csv, etc.)

2) Large tables

If the above items are included with your submission, your manuscript will be returned to you to ensure it follows our Open Research policy guidelines. The “Appendix” and “Data” designations should be considered a simple naming convention used to identify which material will be presented in PDF format (appendices) or spreadsheet format (data). For a definition of a “large table” please see “How do I know if my table is allowed as supporting information?” in our FAQ section.

#### After Acceptance

Following manuscript acceptance, complete data and novel code must be registered in a repository to be made available at the time of publication. ESA Publications staff will verify data and code archiving is complete before releasing files to the publisher. By depositing data and code prior to publication of a manuscript, a permanent link and formal citation can be included in the published paper.

#### Exceptions

Exceptions to this policy are granted only in rare cases as follows and must be fully disclosed by the author at the time of submission.

- Sensitive and confidential information should be redacted as required, including but not limited to precise locations of sampling on private lands, indigenous territory, or sacred sites; locality data for rare, threatened, or endangered species; identity of human subjects.
- In cases in which authors are not the legal data owner and cannot release the data (for example, commercial sources for fish landings), the author should provide sufficient query information so another researcher could seek to obtain the same data, and such limitations must be disclosed at the time of manuscript submission.
- All data on human subjects need to be anonymized to protect the identification of study participants; data from human subjects must be accompanied by an approval statement of the research project by the author’s institutional ethics review committee.

#### Definitions

Raw data and metadata: Raw data are not summarized data in the form of tables of means and standard deviations. Raw data are those used to perform statistical analyses and enable reuse for new or meta-analyses. Data should be provided in non-proprietary, machine-readable formats (e.g., “.csv”). Archived data sets must be accompanied by clear and precise metadata that allow others to readily use files, describing each column of a data set (e.g., locations; elements of experimental design; experimental units; measured variables, methods to measure each variable and units; species abbreviations; etc.).

“Novel” Code: To ensure appropriate code is presented to our readers, any novel code associated with the paper must be provided with the initial manuscript submission for peer review and editorial approval. Novel code is any code that cannot be directly cited and falls under two categories:

- A function or package you have created yourself
- A function or package you have edited to the extent that you can’t simply cite an existing function or package

We require a version of record (the exact version described in the paper that allows a reader to replicate the results presented) provided in the native format of the code file.

This material must be provided in an external repository during the peer review process. We strongly encourage using a repository with a private-for-peer review option, but a public repository posting is also acceptable. If the manuscript is accepted, we require publication of the code in an external repository that permits future updates with versioning control. Novel code in an outside repository must also be properly cited and referenced in the final publication. Code that is not novel (e.g., from a standard statistical package or publicly available model) must be properly cited and referenced.

If your code is not novel, please state this clearly in your Open Research statement. If your statement is ambiguous, the journal’s editorial staff will contact you for further details.

Methods or protocols: Use of known methodology/protocols should be cited appropriately with complete references. Detailed information describing new methodology should be presented in the main text (such as within a “Methods” section) or within an Appendix to be published alongside the manuscript as Supporting Information.

Derived data products: This constitutes data which are collected from database source(s) and collated and/or processed specific to the current research. Complete data source documentation and metadata are required, along with formal citation references. Details regarding data processing steps and assumptions should be clearly defined.

### CHOOSING A REPOSITORY

Data and code must be deposited in a permanent, trusted repository. These guidelines must be followed for both final storage and any material provided as private-for-peer review.

Personal author pages on institutional websites (such as a lab or project page) do not suffice, nor do generic cloud-storage services (such as Google Drive, Box, Sharepoint, Nimbus, or Dropbox), even if these services are licensed through a university or institution.

ESA maintains an extensive list of approved repositories; we encourage you to peruse the General Tips below and contact us with any general questions.

Authors will be responsible for any fees charged by external repositories to comply with the Open Research requirement.

Please note that the repository you select must present the material in English or offer a translation to English.

#### General Tips:

- To guarantee reviewer anonymity, repositories that are used for “private-for-peer review” sharing cannot require a reviewer to log in. Please see our FAQ section (“What are the requirements for “private-for-peer review” storage?”) for more information.
- A trusted repository will assign a DOI or other permanent identifier to the material and guarantees the material to be available in perpetuity.
- When possible, choose a repository that specializes in your scientific field to promote interoperability and reuse.
- Use of a repository run by the institution/affiliation/government agency of the author(s) is encouraged; this staff can be an excellent support system and help authors comply with institute and/or funder requirements.
- The Dryad data repository provides a flexible platform for a wide variety of digital data.
- A no-cost general repository option such as Figshare can be utilized.
- Other examples of permanent repositories include GenBank for DNA sequences, ORNL-DAAC for biogeochemical data, Knowledge Network for Biocomplexity, the NSF Arctic Data Center, and the Environmental Data Initiative (EDI).
- A Zenodo DOI must be obtained for GitHub material to ensure permanency and versioning. Instructions for obtaining a Zenodo DOI can be found here (<https://docs.github.com/en/repositories/archiving-a-github-repository/referencing-and-citing-content> ).

### MECHANICS AND EXAMPLES

#### Online submission form in the ScholarOne system:

At the new submission and revision stages, please consult the numbered list below. After determining the options that apply to you, please do the following: i) Note which options apply to you and disclose the current/future availability of data and code, affirming as many as needed in the Comment Box of the “Open Research” section of the ScholarOne online submission form, AND ii) Add an Open Research statement on the manuscript’s title page that includes any extra requirements for your choice(s) for disclosing the availability of data and code. Submission requirements for each choice are shown in [**bold**].

1. No data were collected for this study (i.e., theoretical, review, opinion, editorial papers).

2. Data are already published and publicly available, with those publications properly cited in this submission. **[If choosing this option, include complete citations for the publicly available data sets you used for your manuscript. You must also include complete citation entries in the References section when possible and cite as appropriate in the main text. If the material was retrieved from an external database that does not use permanent identifiers for datasets, you must provide the necessary query details another qualified researcher would need to obtain the same data you accessed from the external database. Please note: This option is used only for material that is currently publicly available. Do not use this option to refer to the link where your data will eventually be made available if the manuscript is accepted.]**

3. Data are provided as private-for-peer review (shared privately or publicly on a repository). **[If choosing this option, in the Open Research statement you must provide an active link to where the material is currently stored and state the intended repository where data will be permanently archived if the paper is accepted for publication. Data files can NOT be uploaded with your manuscript in the system.]**

4. Data are not yet provided. **[If choosing this option, you must disclose why in the Open Research statement, affirm data will be permanently archived if the paper is accepted for publication, and note the intended repository. Stating data will only be provided upon acceptance of the manuscript is permissible but be advised the Subject-matter Editor or reviewers may request to see this data during review if deemed necessary.]**

5. Data are sensitive and cannot be provided publicly (rare; examples include sensitive species data and human subject data). **[If choosing this option, in the Open Research statement you must fully disclose what data are private and provide complete query and contact details so another qualified researcher can obtain the data. You must provide what data sets you can, with the sensitive data points redacted as necessary. This anonymized data must be provided following the standard archiving guidelines of our Open Research policy. ]**

6. This submission uses novel code, which is provided, per our requirements, in an external repository to be evaluated during the peer review process. **[Novel code files MUST be provided as private-for-peer review in an external archive. A repository with a private-for-peer review option is recommended (such as Dryad or Figshare), but a public repository such as GitHub is acceptable. If choosing this option, your Open Research statement must include the link to the private-for-peer review storage location and state the intended repository where the code will also be permanently archived if the paper is accepted for publication. If your code is not novel, please state this clearly in your Open Research statement.]**

#### Manuscript file:

The manuscript file should include an Open Research statement on the manuscript’s title page that fully describes where readers can access all underlying data and/or code. Statements to the effect of “material are available from the author(s)” are not permissible. Examples of Open Research statements for accepted papers are provided below.

At the submission stage, please consult the numbered list above and include a statement that meets one of these requirements.

For final submissions of accepted manuscripts, this statement must convey complete data archiving details, including data source citations and formal literature citation entries. Note any access restrictions.

#### Examples of text for the “Open Research” statement

**For data and/or code that are currently available in a repository as private-for-peer review:**

Data and/or code are provided as private-for-peer review via the following link: [Link to external storage location]

[Include an active link to the current location where data and/or code can be retrieved]

**For data that are not yet provided, but will be archived upon acceptance:**

Data are not yet provided [Include the reason that data are not yet provided]. Upon acceptance data will be archived in [Include the name of the external repository you will be using].

**For previously published data currently stored in a repository by authors of this research paper:**

Data (Smith et al. 2019) are available from Dryad: [Dryad data set DOI]

[Include complete Smith et al. 2019 data set entry in the Literature Cited and cite as appropriate in the main text. Please note: This option is used only for material that is currently publicly available. Do not use this option to refer to the link where your data will eventually be made available if the manuscript is accepted.]

**For data previously released and published by individuals other than authors of this research paper:**

Data sets utilized for this research are as follows: [citation(s) with DOI/URL data set links]

[Include complete citation entries in the Literature Cited when possible and cite as appropriate in the main text]

**For data retrieved from a public database:**

[Description of specific dataset retrieved] was retrieved from [name of database, including hyperlink]. Query details for retrieving the relevant data are as follows: [provide the necessary query details another qualified researcher would need to obtain the same data].

[Query details should give specific information to direct a reader to download the same data sets you used for your manuscript and should be tailored to the database. Examples of query details include, directions to search pages, search terms used in web forms, the specific names of data sets you used from an available collection, etc. You should not simply state what data in your manuscript came from the database; you should describe how to retrieve it from the database.

If the query details are especially lengthy or complex, you can instead include the specific query details in an appendix and use the following format for the final sentence “Query details are described in [the appendix location where query details are provided]”. This should point to a specific point of your appendix (such as a table or labeled section) where a reader can find the query details.]

**For most theoretical, review, opinion, or editorial papers, and Bulletin Photo Gallery submissions:**

Empirical data were not used for this research.

**(Rare) For data not publicly available, but available to researchers with appropriate credentials:**

Data are not publicly available due to [fill in reasons, such as sensitive species data]. Data can be obtained from [provide complete query and contact details, with license and access restrictions, if any]. Upon acceptance, anonymized data will be archived in [Include the name of the external repository you will be using].

**(Rare) For data that are restricted by commercial, industry, patent, government policies, regulations, and/or laws:**

Data supporting this research are available from [third party source], with [restrictions, including non-disclosure agreements, licensing, other agreements], and are not accessible to the public. [Provide full query and contact process details for other researchers to gain access.]

NOTE: If your data are in either of the “Rare” statement categories, the Subject-matter Editor will be asked to determine if the statement provides the detail necessary to meet ESA guidelines.

### Frequently Asked Questions

This section is a living document, and we intend to expand the list. Additional questions can be submitted to esajournals@esa.org

**Q: What are the advantages of posting my data and code on a public repository?**

A: Advantages of using a permanent repository include:

- Visibility: Making your data/code available online (and linking it to the journal publication) provides a new pathway for others to learn about your work.
- Citability: All data/code you deposit will receive a persistent, resolvable identifier that can be used in a citation.
- Workload reduction: If you receive individual requests for data/code, you can simply direct them to files in the archive.
- Quality control: storage of data in a permanent public repository encourages complete transparency and discourages the falsification of data and other fraudulent publication practices in ESA journals.
- Preservation: Your data/code files will be permanently and safely archived in perpetuity.
- Impact: You will garner citations for both the research paper and data/code product through the reuse of your data/code.
- Potential for greater collaboration: Other scientists searching for data to perform meta-analyses or develop new hypotheses may involve you as a collaborator and author.

**Q: Do I need to archive my data prior to manuscript submission?**

A: Only for “Data Papers” and “Statistical Reports” in Ecology. For other manuscript types and journals, it is not required at submission but is strongly encouraged. If you are depositing data in a repository at the manuscript submission stage, we suggest keeping it “private for peer review” and this data deposit should not specify the journal since the paper is not formally accepted. Except for “Data Papers” and “Statistical Reports” in Ecology, at the submission stage we only require confirmation of understanding of the policy and mention of the intended repository for verification purposes.

**Q: Do I need to provide my novel code at the time of manuscript submission?**

A: Yes, novel code is required in order to be evaluated. Just like the main manuscript and other files, this material is provided to reviewers as part of the reviewing package. Novel code must be made accessible via an external archive during all stages of the review process. A repository with a private-for-peer review option is recommended (such as Dryad or Figshare), but a public repository such as GitHub is acceptable.

**Q: How should I prepare my code?**

A: Best practices guidelines advise authors to use a scriptable statistical environment, generating statistical code that is sufficiently complete to allow replication of tables, graphs, and statistical analyses reported in the original publication and perform new or meta-analyses. Every number, every P value, and every graph requires formal archiving of the original script that generated it. In cases in which published results are challenged or questioned, these scripts will be used to reconstruct the published analyses, which is the critical first step towards resolving the dispute.

**Q: What are the requirements for sharing novel code (required) and data (not required) during the review process?**

A: Authors are not required to provide data as during the review process at initial submission but it is strongly encouraged. Novel code, however, must be provided during the review process.

If an author provides material during the review process, the repository must guarantee reviewer anonymity. A repository cannot require a reviewer to log in to access the material. Personal author pages on institutional websites (such as a lab or project page) also do not suffice, nor do generic cloud-storage services (such as Google Drive, Box, Sharepoint, Nimbus, or Dropbox), even if these services are licensed through a university or institution.

The best practice is to use a repository that provides a direct link to the “private-for-peer review” material with no log-in required, such as Figshare or Dryad. A public-facing repository such as GitHub will also be acceptable.

**Q: What do you mean by “private-for-peer review”?**

A: “Private-for-peer review” refers to the process of storing material temporarily as private during the review process, with access provided by a direct anonymous link to the material. For material that is provided during the review, private-for-peer review options are recommended (such as Dryad or Figshare), but a public repository where material can be viewed anonymously, but it is not stored privately (such as GitHub) is acceptable. We do not allow generic cloud-storage services for private-for-peer review or for final archival of material, even if these services are licensed through a university or institution.

**Q: What are the requirements for “private-for-peer review” storage?**

A: Authors are not required to provide data as “private-for-peer review” at initial submission but it is strongly encouraged. Novel code, however, must be provided as private-for-peer review.

If an author provides material as “private-for-peer review”, the repository must guarantee reviewer anonymity. A repository cannot require a reviewer to log in to access the material as “private-for-peer review”. Personal author pages on institutional websites (such as a lab or project page) also do not suffice, nor do generic cloud-storage services (such as Google Drive, Box, Sharepoint, Nimbus, or Dropbox), even if these services are licensed through a university or institution.

The best practice is to use a repository that provides a direct link to the “private-for-peer review” material with no log-in required, such as Figshare or Dryad. A public-facing repository such as GitHub be acceptable.

**Q: What happens if I have archived my data and my paper is not accepted by the journal?**

A: Your data on the repository remains there and can be linked to a new submission at a different journal. Your data deposited on a repository should not specify a related journal publication until the paper is formally accepted.

**Q: Can I provide my data or code after my accepted manuscript is released online?**

A: No, data and code links must be verified and included in the final file sent to the publisher. Authors will need to archive data/code as part of the final submission process. Manuscript files will not be sent to the publisher for typesetting until this requirement is met.

**Q: Can I embargo the data or code associated with my accepted manuscript in order to release the data or code at a later time?**

A: No, we do not permit any kind of data or code embargo following manuscript acceptance. Related data and code must be publicly available before the final manuscript is sent to the publisher for typesetting. The desire of authors to control additional research with these data and/or code shall not be grounds for withholding material.

**Q: What should I name my data or code package in the external repository?**

A: To avoid confusion and possible errors, data and code packages should not be named the exact same name as your manuscript title. Use a descriptive name for data and code packages which meets the guidelines of the repository. A suggested format is “Data from: [manuscript title]” or “Code for: [manuscript title]”. Do include a reference to your related ESA journal publication in data and code deposits.

Individual files in your data or code deposit should be given unique and descriptive file names that can be referred to when necessary (for example: “Ungulate_geospatial_data.xlsx”). Please do not use the format “Data S#”, “Appendix S#”, “Table S#”, or any similar “Item S#” name for the material being placed on a data or code repository, as this naming convention is used by the publisher for supporting information hosted on the journal platform and can result in confusion during file processing and for readers at all stages.

**Q: What repository should I use if I have a very large dataset?**

A: In previous cases, authors with very large datasets in the 100s of GB range have successfully used Figshare or Knowledge Network for Biocomplexity to meet the requirements of our Open Research policy.

**Q: How do I know if my table is allowed in an appendix as supporting information?**

A: Appendix tables cannot be provided as spreadsheet files. If your table requires a spreadsheet format, it must be provided under the Open Research policy. Appendix tables will be published in PDF format and should be presented in the clearest and most readable format possible, with font sizing at 8-pt or larger. Appendix tables can be no longer than two pages. If an appendix table is too large, staff may require you remove it and provide it as data under the Open Research policy for ease of re-use. Exception: “Data Papers” in Ecology should provide data in spreadsheet format.

**Q: Will ESA help me craft my Open Research statement?**

A: Please make every effort to review and follow the statement examples provided in the “Mechanics and Examples” section. After you have submitted your manuscript, your statement will be checked by the journal’s editorial staff and they will request clarifications if necessary. Specific questions can be directed to the journal’s editorial staff .

**Q: The repository requires the journal publication DOI before releasing the data. How can I obtain the DOI for my ESA journal paper to link to it?**

A: The publisher will assign the DOI shortly after their receipt of the files, and most data repositories can amend the data release to include this detail when requested to do so. Contact the journal’s editorial staff if your paper is accepted and your repository is not able to accommodate this.

**Q: ESA will link the paper to the data set, but how can I link the data set to the paper?**

A: Please work with your repository to link the data set back to the journal release. This requires some finesse because the journal article will not yet be released.

**Q: I would like to deposit data in Dryad. Does your journal provide manuscript information to them?**

A: Yes! Please let us know you will be using Dryad when uploading your manuscript submission, and we can simplify your data submission process with Dryad. Detailed Dryad submission instructions will be provided at manuscript acceptance.

**Q: Are there fees associated with depositing data?**

A: Authors are responsible for fees associated with data deposits. Authors of Ecological Monographs contributions that choose to deposit data in Dryad will be assessed a reduced fee. Repository fees typically range from $0 to $120 for most data sets, with structured, incremental fee increases for very large deposits (these are generally structured by ranges of gigabytes). Sites like Figshare are a no-cost option, as are many university library repositories.

**Q: When referring to the published paper and/or data set, should I cite the paper, the data set, or both?**

A: Both items are considered research output, and both should be cited as appropriate.

**Q: What precautions should be taken with sensitive human subject data?**

A: If you work with human subjects, be sure to follow all relevant guidelines for the protection of personally identifying information (PII) as specified in your Institutional Review Board (IRB) application and approval. Data need to be sufficiently anonymous so that identity cannot be surmised from the data set.

**Q: What additional resources are available?**

A: We encourage authors to explore the FAIR principles and Powers and Hampton (2019) in Ecological Applications (Open Access: https://doi.org/10.1002/eap.1822). Authors, editors, reviewers, and readers are encouraged to reach out to the journal’s editorial staff with any questions for manuscript preparation or regarding previously published materials.

# 8. Global Change Biology

<https://onlinelibrary.wiley.com/page/journal/13652486/homepage/forauthors.html>

## Data Sharing and Data Accessibility

Global Change Biology requires, as a condition for publication, that the data supporting the results in the paper be archived in an appropriate public repository. Data archiving must be completed before files will be sent to the publisher. Both primary and secondary data needs to be publicly available as a condition for publication.

Manuscript files will not be sent to the publisher unless the following three criteria are met:

- A data availability statement which provides information about where the research data and other artifacts supporting the results reported in the paper can be found must be submitted. Links to the repository where the dataset(s) are publicly archived and DOIs must be included. A list of standard templates for the text for the ‘Data Availability Statement’ is available here (<https://authorservices.wiley.com/author-resources/Journal-Authors/open-access/data-sharing-citation/data-sharing-policy.html#standardtemplates> ).
- Data must be cited within the text in the in the Materials and Methods section.
- Data must be included as a formal citation in the reference section. More detail on how to do this can be found here (<https://authorservices.wiley.com/author-resources/Journal-Authors/open-access/data-sharing-citation/data-citation-policy.html> ).

Additional information:

- Data must be archived in an appropriate public archive, such as NCBI, Gene Expression Omnibus, TreeBASE, Dryad, FigShare, or your own institutional or funder repository. Visit re3data.org to find additional registered and certified data repositories.
- Data supporting a paper’s results and conclusions must be archived with sufficient details and metadata so that a third party can interpret them correctly. Papers with exemplary data and code archiving are more valuable for future research.
- Authors are encouraged to include code for statistical computing in the online supplementary material.
- Embargos may be granted in exceptional instances at the discretion of the Editor, such as for human subject data, the location of endangered species, or long-term ecological data sets.
- For additional guidelines on data deposition best practice, please review the FORCE11 Data Citation Principles

(<https://www.force11.org/datacitationprinciples> ).

Manuscripts that include modelling:

- Manuscript must include full equations and parameters in the text, supplementary files, or cited publicly accessible repository. This information must also be included in the Data Availability statement.
- Global Change Biology mandates the sharing of code, software and documentation supporting the results in the paper following acceptance. It may be archived in an appropriate public repository (e.g., Zenodo). This information must be included in the and listed in the Data Availability Statement.

Please contact the editorial office if you have any questions.

# 9. Methods in Ecology and Evolution

<https://besjournals.onlinelibrary.wiley.com/hub/journal/2041210X/author-guidelines>

## Code and data review

Code and data should be available at submission in an anonymised form. Authors can upload the file(s) for editors and reviewers as a zip, CSV, txt, md or rmd file (or similar) or use any archive that allows private for peer review (such as Dryad or Zenodo), see more information here. For more complex projects, a zipped folder containing all code required is a better option than uploading all individual files separately, and can easily be obtained from many code repositories for example GitHub. For a quick guide on anonymising your code, please see the video below.

We encourage authors to check that files do not include identifying information (such as their name in the script, metadata or README).

Authors of accepted articles will be able to update this to a formal Data Availability Statement at acceptance.

### Making your code anonymous for peer review (YouTube video)

<https://www.youtube.com/watch?v=DgnF5tDRGTY>

## Data Availability

To enable readers to locate archived data from papers, we require that authors list the database and the respective accession numbers or DOIs for all data from the manuscript that has been made publicly available. For example, “Data available from the Dryad Digital Repository http://dx.doi.org/10.5061/dryad.41qh7 (Kiere & Drummond 2016).” When a DOI is available for the data, the full data citation should also be given in the reference list (see below). For further information see our data archiving policy (<https://besjournals.onlinelibrary.wiley.com/hub/data_archiving_policy> ).

## Data sources (where appropriate)

Authors of submissions that use data from multiple published sources (e.g. if the paper describes a meta-analysis) should cite these data sources in the main text of the manuscript or include a separate Data Sources section as appropriate. This ensures that these references are fully indexed and their authors are given proper citation credit.

Data sources can be cited in the “Materials and methods” or in the “Data availability” sections. If a large number of data sources are used, instead of citing the sources individually, a separate list should be provided after the literature reference list under the heading “Data sources”. The Material and methods section should then refer to this section, i.e. “A list of data sources used in the study are provided in the Data sources section.”

Data from articles published in journals or data with a DOI should follow the normal journal citation format. Citation of datasets without a DOI is permitted, provided the data repository meets the standards set out in our Data Archiving policy. They should be formatted as above but should provide the permanent repository link and accession number for the data.

## British Ecological Society data archiving policy

<https://besjournals.onlinelibrary.wiley.com/hub/data_archiving_policy>

Data are important products of the scientific enterprise, and they should be preserved and usable for decades in the future. The British Ecological Society thus requires, as a condition for publication, that all data supporting the results in papers published in its journals are archived in an appropriate public archive offering open access and guaranteed preservation. For theoretical papers the underlying model code must be archived.

The data underlying all the results presented in the paper must be archived in a format that allows a third party to reproduce the data. The archived data must allow each result in the published paper to be recreated and the analyses reported in the paper to be replicated in full to support the conclusions made. Authors are welcome to archive more than this, but not less.

Authors may elect to have the data made publicly available at time of publication or, if the technology of the archive allows, may opt to embargo access to the data for a period of up to a year after publication. Exceptions, including longer embargoes, may be granted only in exceptional circumstances at the discretion of the editor, especially for sensitive information such as confidential social data or the location of endangered species.

All papers must have a data archiving statement and data sources must be cited in the reference list (see submission guidelines).

### Suitable archives

Authors are free to use any repository and are encouraged to use a repository that is best suited to their data and is most useful to the ecological community likely to access their data. The data repository used must guarantee preservation of the data and the data itself must be freely available (users must not have to request access to obtain the data). Data must not be uploaded as Supporting Information of an article. A repository that issues a persistent identifier such as a DOI or provides accession numbers is preferable so specific data sets can be referenced. Below is a list of commonly used archives for ecological data. For software source code, we recommend the use of a public software repository with version control for the ongoing maintenance of software packages. Other archives are checked in house and authors may be contacted if the repository’s polices are not clear.


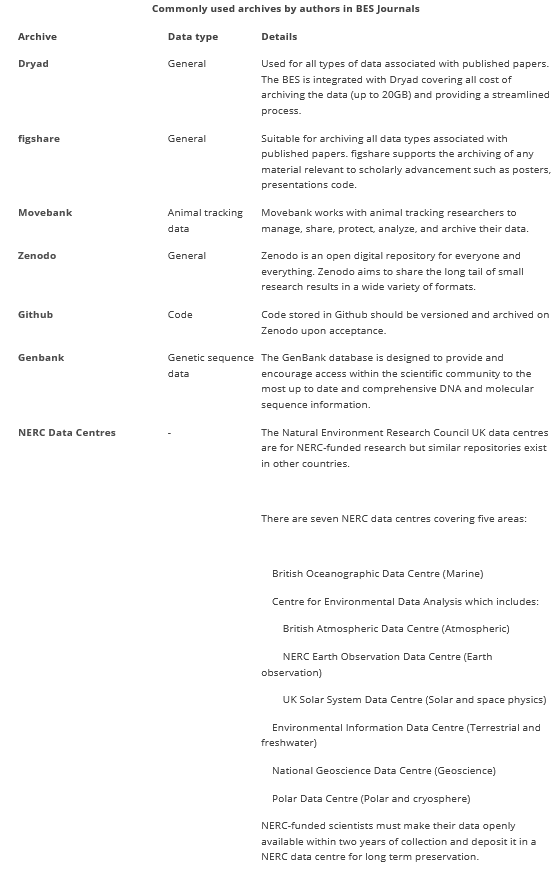


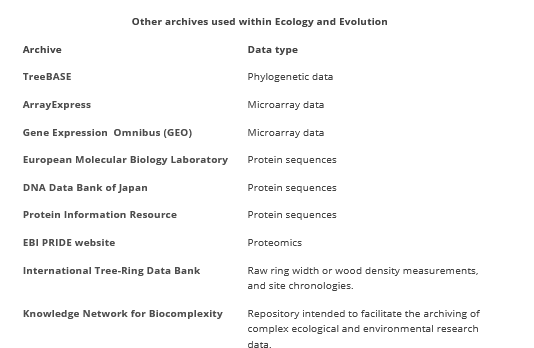


### Third-party data, embargoes and waivers

**Third-party data**

It is the author’s responsibility to seek permission to archive the relevant data so that the data are publicly available. In cases where there is proprietary data which is not owned by any of the authors and permission to archive the data is not granted, evidence of any refusals (e.g. email exchange) by third parties must be sent to the Journal Editorial Office. Where permissions are not granted for archiving data held in restricted databases or owned by private organisations the location of the data must be included in the ‘Materials and methods’ section of the paper including contact details for the organisation where the data is held . These details must also be included in the ‘Data Availability Statement’ below the ‘Acknowledgements’. Where an author works for an institution that owns the data, the data associated with a paper must be archived.

**Embargoes**

At the time of deposition, authors may choose to embargo their data for up to 12 months after online publication. Longer embargoes may be granted at the discretion of the editors. These embargoes will provide the data creators the opportunity of first use of the data. Embargoed data must be deposited in an archive prior to publication but can be restricted from public view for the duration of the agreed embargo.

**Sensitive data and waivers**

For sensitive data relating to endangered species or protected locations, authors should transform locality details or provide an anonymised version of the dataset whenever possible. In situations when endangered species or protected locations cannot be transformed, when data access is politically or culturally-sensitive or when datasets include sensitive social data/information, editors may waive the archiving requirement. Authors must provide a short explanation in the Data Availability Statement when the archiving requirement has been waivered.

### FAQs

**Why does data associated with papers published in BES journals have to be archived?**

At present most ecological data are lost to science as they are stored unreliably or discarded by scientists when they look at new research areas, change careers, retire or die. To promote the preservation and fuller use of ecological data the BES requires the archiving of data associated with papers published within its journal portfolio. Data reported in papers are invaluable to science even after the paper has been published for uses in meta-analyses, new research or quality control. By requiring the archiving of data referred to in the peer-reviewed literature each journal can play a role in helping researchers meet the requirements of funding bodies and support the long-term research that advances the science of ecology.

**Why should I share my data?**

It is increasingly common for funders and publishers to mandate data sharing wherever possible. Further benefits of sharing data include:

- Increasing the impact and visibility of research
- Encouraging collaborations and partnerships with other researchers
- Maximising transparency and accountability
- Encouraging the improvement and validation of research methods
- Reducing the cost of duplicating data collection
- Advancing science by letting others use data in innovative ways
- Data can be cited in order to give credit to the data creator

**The data associated with this paper have already been archived. Do they need to be archived again?**

If data have been previously archived then they should not be archived again. The original archive DOI or reference should be used as the source of the data.

**What format does the data need to be in?**

We do not recommend a single file format; rather the data should be archived in a format that follows community standards and is most useful to the community accessing the data. Non-proprietary files, such as ASCII text files and CSV are most appropriate, as this is more likely to ensure the files are readable in the future. Data should be archived in a format that is easily extracted from the files provided (e.g., by using CSV rather than PDF).

The necessary metadata must be provided with the data set so that the data set is understandable on its own. For example, this should include definitions for all terms, variables, names, row and column headings and precise locations. This is best provided in an accompanying file archived with the data. Data in their most raw form, such as videos, field notebooks or sequencing trace files are not required to be archived unless they are integral to recreating the results in the paper. For more information on effective data management, please click here for our data management booklet.

**Once archived, who will own the data?**

Although data will be publicly accessible, they will remain the intellectual property of the original collectors of the data. Any re-use of archived data must be referenced appropriately. Please see the terms and conditions provided by your chosen archive for details on their policies for data reuse and citation.

**I have a large number of data sources, where should this information be included?**

Authors of submissions that use data from multiple published sources (e.g. if the paper describes a meta-analysis) are encouraged to cite these data sources in the main text of the manuscript. This ensures that these references are fully indexed and their authors are given proper citation credit. For more details see citations to data sources.

# 10. Molecular Biology and Evolution

<https://academic.oup.com/mbe/pages/General_Author_Guidelines>

**Data and Resource Availability:** MBE requires that all new data are publicly available upon publication. Any published manuscript reporting new data must contain a clear data availability statement before the Acknowledgements. This section will report accession numbers for public databases for all newly-reported sequences and structural coordinates. It should also list all resources - including vcfs, alignments, computational pipelines and other resources - that have been made available in public repositories like github, Data Dryad, Zotero and Figshare. All data in public repositories should have clear descriptions, perhaps in an accompanying read.me file.

# 11. Molecular Ecology

<https://onlinelibrary.wiley.com/page/journal/1365294x/homepage/forauthors.html>

## Data Storage and Documentation

Molecular Ecology requires, as a condition for publication, that the data supporting the results in the paper will be archived in an appropriate public repository. Essential metadata such as spatio-temporal data should be archived and linked to associated genetic data when organisms have been collected from natural, wild, or domesticated populations (for example, spatio-temporal data may not be relevant for laboratory strains or some experimental systems). Whenever possible the scripts and other artefacts used to generate the analyses presented in the paper should also be publicly archived. Embargos may be granted in exceptional instances at the discretion of the Editor, such as for human subject data, the location of endangered species, or long-term ecological data sets.

We require that authors include a ‘Data Accessibility and Benefit-Sharing’ section after the References (see 'Preparing the Submission' Section for details). This section must be present at initial submission, and data archiving must be completed before final acceptance. Alongside this, we ask that authors also cite their data according to the Force 11 Data Citation Principles (<https://www.force11.org/datacitationprinciples> ). More detail on how to do this can be found here: <https://authorservices.wiley.com/author-resources/Journal-Authors/open-access/data-sharing-citation/data-citation-policy.html>.

Data are important products of the scientific enterprise, and they should be preserved and usable for decades in the future. As such, Molecular Ecology requires authors to archive the data supporting their results and conclusions along with sufficient details so that a third party can interpret them correctly. Papers with exemplary data and code archiving are more valuable for future research, and, all else being equal, will be given higher priority for publication.

**Good Practice:**

- Data should be archived in an appropriate public archive, such as NCBI, Gene Expression Omnibus, TreeBASE, Dryad, FigShare, the Knowledge Network for Biocomplexity, your own institutional or funder repository, or as Supporting Information on the Molecular Ecology web site. Raw sequence data (including transcriptome sequences) should be archived in an internationally approved genomic repository such as NCBI or the European Nucleotide Archive.
- To maximise the reproducibility and reusability of data, essential metadata such as spatio-temporal data should be archived with clear and permanent links to associated genetic data. Molecular Ecology encourages authors to use GEOME for improved data archiving. More information on this resource can be found here: <https://onlinelibrary.wiley.com/doi/10.1111/1755-0998.13283> . Authors submitting to the journal who use GEOME for data archiving should use the preformatted ‘Molecular Ecology/Molecular Ecology Resources’ template.
- Upon acceptance, the Data Accessibility statement must be completed including database and information such as accession numbers or DOI (as available) for all data from the manuscript.
- The utility of archived data is greatly enhanced when the scripts and input files used in the analyses are also made available. Given that scripts may be a mix of proprietary and freely available code, their deposition is not compulsory, but we nonetheless strongly encourage authors to make these scripts available whenever possible.
- Reproducibility is key for maintaining trust in science. Since R markdown or Jupyter notebooks are an important step towards more reproducibility, Molecular Ecology encourages authors to include them in the online supplementary material.
- Software and documentation may be made accessible from a long-term server (e.g., GitHub), however, at least a snapshot of these resources must be posted on Dryad, CRAN, or similar academic/publishing archiving sites, with a link to a long-term server where software development and future releases can be found such that continued access to these resources is ensured.
- Data tables should be archived in a re-usable file format e.g. .xlsx files.
- Whitlock et al. (2010) (<https://www.journals.uchicago.edu/doi/full/10.1086/650340> ), state that accurate interpretation of data will likely "require a short additional text document, with details specifying the meaning of each column in the data set. The preparation of such shareable data sets will be easiest if these files are prepared as part of the data analysis phase of the preparation of the paper, rather than after acceptance of a manuscript."
- For additional guidelines on data deposition best practice, please visit <http://datadryad.org/depositing>

If you have any questions about the data archiving requirements, please email molecol@wiley.com to request further information.

# 12. Molecular Ecology Resources

<https://onlinelibrary.wiley.com/page/journal/17550998/homepage/forauthors.html>

## Data Accessibility and Benefit-Sharing Section

Molecular Ecology Resources requires authors to include a Data Accessibility and Benefit-Sharing section in their manuscript, located beneath the references. The Data Accessibility and Benefit-Sharing section should be divided into two distinct statements: a Data Accessibility Statement and a Benefit-Sharing Statement, each with their own sub-heading. Please see the information below for guidance on what should be included in each statement. A Data Accessibility and Benefit-Sharing section that contains only a Data Accessibility Statement will be permitted in cases where there are no benefits to report.

### Data Accessibility Statement

Molecular Ecology Resources requires, as a condition for publication, that the data supporting the results in the paper will be archived in an appropriate public repository. Essential metadata such as spatio-temporal data should be archived and linked to associated genetic data when organisms have been collected from natural, wild, or domestic populations (for example, spatio-temporal data may not be relevant for laboratory strains or some experimental systems). Whenever possible the scripts and other artefacts used to generate the analyses presented in the paper should also be publicly archived. Exceptions may be granted at the discretion of the editor, especially for sensitive information such as human subject data or the location of endangered species.

Authors are required to archive their data in a publicly accessible repository such as Dryad, FigShare, GenBank, etc. (not a laboratory homepage).

Upon submission, the Data Accessibility and Benefit-Sharing section must include a Data Accessibility Statement, but this can describe curation plans prior to data having been thus archived.

Upon acceptance, data must be archived and the Data Accessibility Statement completed including database and information such as accession numbers or DOI (as available) for all data from the manuscript before the manuscript can be published.

Note: if data, scripts, or other artefacts used to generate the analyses presented in the paper are available via a publicly available data repository, authors should include a reference to the location of the material within their paper.

**Example text for Data Accessibility Statement:**

Genetic data:

- Raw sequence reads are deposited in the SRA (BioProject XXX)
- Individual genotype data are available on DataDryad (XXXX)
- Unique haplotype data are deposited to NCBI Nucleotide Database (XXXX)

Sample metadata:

- Metadata are also stored in the SRA (BioProject XXX) using the XXXX MIxS package XXXX

OR

- Metadata can be found in GEOME (DOI XXX; including georeferences in decimal degrees and date/month/year of sampling event)

OR

- Related metadata can be found in XXX (including georeferences in decimal degrees and date/month/year of sampling event) and XXX provides unique sample identifier tags that can be matched to both the deposited genetic data and deposited metadata (for haplotypes, individual sample identifiers and their corresponding haplotype)

### Benefit-Sharing Statement

Molecular Ecology Resources additionally requires, as a condition for publication, that the research described in the publication complies with relevant national laws implementing the Convention on Biological Diversity and Nagoya Protocol agreements. Authors will be required to make an affirmative statement during the submission process as to compliance with national laws, if applicable.

Molecular Ecology Resources also encourages authors to include a Benefit-Sharing Statement within their Data Accessibility and Benefit-Sharing section. The Benefit-Sharing Statement should disclose benefits generated commensurate with the Nagoya Protocol. For further information on the scope of benefits recognized under the Nagoya Protocol, see the link to the Nagoya Protocol Annex at <https://www.cbd.int/abs/text/articles/?sec=abs-37> .

We recognize in some cases the Nagoya Protocol is not applicable or that there are no benefits to report, which is why the reporting of benefits is not mandatory.

**Example Text for Benefit-Sharing Statement:**

"Benefits Generated: A research collaboration was developed with scientists from the countries providing genetic samples, all collaborators are included as co-authors, the results of research have been shared with the provider communities and the broader scientific community (see above), and the research addresses a priority concern, in this case the conservation of organisms being studied. More broadly, our group is committed to international scientific partnerships, as well as institutional capacity building.

"Benefits Generated: We consulted with the indigenous community providing the biodiversity resources and hired members of a local Hunters and Trappers Association to help with biodiversity assessments, including collections of canid fecal samples for diet analysis based on DNA metabarcoding, and local knowledge concerning changes in prey communities over time. The contributions of all individuals to the research, including indigenous hunters, are described in the METHODS and ACKNOWLEDGEMENTS, and a research report has been provided to the relevant indigenous community council, as well as to the territorial government. The research addresses a priority concern regarding an apparent shift in the prey of canids from wild to domesticated animals. Lastly, as described above, all data have been shared with the broader public via appropriate biological databases.”

“Benefits Generated: Benefits from this research accrue from the sharing of our data and results on public databases as described above.”

Manuscripts lacking a Data Accessibility and Benefit-Sharing section will not be passed through to an editor. Please note that reviewers will be asked to comment on the completeness of this section. If you have any questions about the Data Accessibility and Benefit-Sharing section, please email molecol@wiley.com to request further information.

### Open Data Badges

In partnership with the non-profit Center for Open Science (COS) (<https://www.cos.io/> ), Molecular Ecology Resources invites all submitting authors to apply for an Open Data Badge (<https://authorservices.wiley.com/open-research/open-practices/index.html> ). The Open Data Badge recognizes researchers who make their data publicly available, providing sufficient description of the data to allow researchers to reproduce research findings of published research studies. Applying and qualifying for Open Data Badges is not a requirement for publishing with Molecular Ecology Resources but these badges are further incentive for authors to participate in the Open Research movement and thus to increase the visibility and transparency of their research. For an example of an article with an Open Data Badge, click here (<https://onlinelibrary.wiley.com/doi/10.1111/1755-0998.13459> ).

There are, of course, circumstances in which it is not possible or advisable to share data publicly, especially for sensitive information such as human subject data or the location of endangered species. In these cases, the authors will have the opportunity to provide an explanation of such circumstances. The information the authors provide will be included in the article’s Open Research note.

NOTE: If you are applying for an Open Data Badge for the first time at a revision or resubmission stage, please email the Managing Editor at molecol@wiley.com.

## Data Storage and Documentation

Molecular Ecology Resources requires, as a condition for publication, that the data supporting the results in the paper will be archived in an appropriate public repository. Essential metadata such as spatio-temporal data should be archived and linked to associated genetic data when organisms have been collected from natural, wild, or domesticated populations (for example, spatio-temporal data may not be relevant for laboratory strains or some experimental systems). Whenever possible the scripts and other artefacts used to generate the analyses presented in the paper should also be publicly archived. Embargos may be granted in exceptional instances at the discretion of the Editor, such as for human subject data, the location of endangered species, or long-term ecological data sets.

We require that authors include a ‘Data Accessibility and Benefit-Sharing’ section after the References (see 'Preparing the Submission' Section for details). This section must be present at initial submission, and data archiving must be completed before final acceptance. Alongside this, we ask that authors also cite their data according to the Force 11 Data Citation Principles. More detail on how to do this can be found here: <https://authorservices.wiley.com/author-resources/Journal-Authors/open-access/data-sharing-citation/data-citation-policy.html>

Data are important products of the scientific enterprise, and they should be preserved and usable for decades in the future. As such, Molecular Ecology Resources requires authors to archive the data supporting their results and conclusions along with sufficient details so that a third party can interpret them correctly. Papers with exemplary data and code archiving are more valuable for future research, and, all else being equal, will be given higher priority for publication.

**Good Practice:**

- Data should be archived in an appropriate public archive, such as NCBI, Gene Expression Omnibus, TreeBASE, Dryad, FigShare, the Knowledge Network for Biocomplexity, your own institutional or funder repository, or as Supporting Information on the Molecular Ecology Resources web site. Raw sequence data (including transcriptome sequences) should be archived in an internationally approved genomic repository such as NCBI or the European Nucleotide Archive.
- To maximise the reproducibility and reusability of data, essential metadata such as spatio-temporal data should be archived with clear and permanent links to associated genetic data. Molecular Ecology Resources encourages authors to use GEOME for improved data archiving. More information on this resource can be found here: https://onlinelibrary.wiley.com/doi/10.1111/1755-0998.13283. Authors submitting to the journal who use GEOME for data archiving should use the preformatted ‘Molecular Ecology/Molecular Ecology Resources’ template.
- Upon acceptance, the Data Accessibility statement must be completed including database and information such as accession numbers or DOI (as available) for all data from the manuscript.
- The utility of archived data is greatly enhanced when the scripts and input files used in the analyses are also made available. Given that scripts may be a mix of proprietary and freely available code, their deposition is not compulsory, but we nonetheless strongly encourage authors to make these scripts available whenever possible.
- Reproducibility is key for maintaining trust in science. Since R markdown or Jupyter notebooks are an important step towards more reproducibility, Molecular Ecology Resources encourages authors to include them in the online supplementary material.
- Software and documentation may be made accessible from a long-term server (e.g., GitHub), however, at least a snapshot of these resources must be posted on Dryad, CRAN, or similar academic/publishing archiving sites, with a link to a long-term server where software development and future releases can be found such that continued access to these resources is ensured.
- Data tables should be archived in a re-usable file format e.g. .xlsx files.
- Whitlock et al. (2010), state that accurate interpretation of data will likely "require a short additional text document, with details specifying the meaning of each column in the data set. The preparation of such shareable data sets will be easiest if these files are prepared as part of the data analysis phase of the preparation of the paper, rather than after acceptance of a manuscript."
- For additional guidelines on data deposition best practice, please visit http://datadryad.org/depositing.

If you have any questions about the data archiving requirements, please email molecol@wiley.com to request further information.

# 13. Nature Ecology & Evolution

<https://www.nature.com/natecolevol/submission-guidelines/registeredreports>

## Author Guidelines for Registered Reports

A Registered Report is a form of empirical article offered at Nature Ecology & Evolution in which the methods and proposed analyses are pre-registered and peer reviewed prior to research being conducted. High quality protocols are provisionally accepted for publication before data collection commences. This format is designed to minimize publication bias and research bias in hypothesis-driven research, while also allowing the flexibility to conduct exploratory (unregistered) analyses and report serendipitous findings.

Overview of the process

Initial submissions will include a description of the key research question(s) and background literature, hypotheses, experimental procedures, analysis pipeline, a sampling plan (statistical power analysis or Bayesian equivalent), and pilot data (where applicable). Please use this template to prepare your Stage 1 submission.

Initial submissions will be assessed by the editorial team for strength of scientific advance and suitability for a broad, multidisciplinary audience. Those that meet our criteria will then be sent for in-depth peer review (Stage 1). Following review, the article will be either rejected, revised, or accepted in principle for publication. After acceptance in principle (AIP), the authors will proceed to conduct the study, adhering exactly to the peer-reviewed procedures. When the study is complete the authors will submit their finalised manuscript for re-review (Stage 2) and will upload their raw data, study materials, and computer code (if relevant) to a publicly accessible file-sharing service. Pending further peer review to ensure a sensible interpretation of the findings, the manuscript will be published regardless of the significance or direction of the results.

### Stage 1: Initial manuscript submission

Nature Ecology & Evolution aims to publish research of outstanding significance. For this reason, the editors select only the most scientifically promising manuscripts for in-depth peer review. Stage 1 submissions should include the manuscript (details below) and a brief cover letter. Authors are welcome to submit presubmission enquires for advice on the likely suitability of a study as a Registered Report. However, please note that we cannot commit to sending a manuscript for in-depth review until a complete Stage 1 submission has been evaluated by the editors.

**The Stage 1 cover letter should include:**

- A brief scientific case for consideration. The journal aims to publish research that represents a significant scientific advance and is of relevance to a broad, multidisciplinary audience. High-value replication studies are welcome in addition to novel studies.
- A statement confirming that all necessary support (e.g. funding, facilities) and approvals (e.g. ethics) are in place for the proposed research. Note that manuscripts will be generally considered only for studies that are able to commence immediately; however authors with alternative plans are encouraged to contact the journal office for advice.
- An anticipated timeline for completing the study if the initial submission is accepted.
- A statement confirming that the authors agree to share their raw data, any digital study materials, and computer code (if relevant) for all published results.
- A statement confirming that, following Stage 1 acceptance in principle, the authors agree to register their approved protocol on a recognized repository, either publicly or under private embargo until submission of the Stage 2 manuscript.
- A statement confirming that if the authors later withdraw their paper, they agree to the Journal publishing a short summary of the pre-registered study under a section Withdrawn Registrations.

### Manuscript preparation guidelines - Stage 1

Initial Stage 1 submissions should include the sections listed below. More detail for the contents of each section can be found in the template document (<https://www.nature.com/documents/np-nee-template-stage1.pdf> ).

Introduction

Methods

- Ethics information (if relevant for studies with humans or animals)
- Pilot data (optional)
- Study design (with information on controls, randomization, and blinding, if relevant)
- Sampling plan (containing a power analysis where possible)
- Analysis plan (full details of data preprocessing and analytical steps)

Data Availability

Code Availability

References

Figures (optional but encouraged)

Table of hypotheses (mandatory)

### Stage 2: Full manuscript submission

Once the study is complete, authors prepare and resubmit their manuscript for full review, with the following additions:

**The Stage 2 cover letter must confirm:**

- That the manuscript includes a link to the public archive containing anonymized study data, digital materials/code and the laboratory log. Within the manuscript, this information should appear in two separate sections, entitled ‘Data availability’ and ‘Code availability’.
- That the manuscript contains a link to the approved Stage 1 protocol in a standalone section entitled ‘Protocol Registration’.
- That, for primary Registered Reports, no data for any pre-registered study (other than pilot data included at Stage 1) was collected prior to the date of AIP. For secondary Registered Reports, authors should confirm that no data (other than pilot data included at Stage 1) was subjected to the pre-registered analyses prior to AIP.

**Submission of raw data:**

- Anonymized raw data, processed data, and computer code (if relevant) must be made freely available in a public repository. Authors are free to use any repository that renders data and materials freely and publicly accessible and provides a digital object identifier (DOI) to ensure that the data remain persistent, unique and citable.
- Data files ideally will be time stamped to show that data was collected after AIP and not before. Other than pre-registered and approved pilot data, no data acquired prior to the date of AIP is admissible in the Stage 2 submission. Raw data must be accompanied by guidance notes or meta-data to assist other scientists in replicating the analysis pipeline. Authors are also expected to upload any relevant analysis scripts and other experimental materials that would assist in replication.
- Supplementary figures, tables, or other text (such as supplementary methods) should be included as standard supplementary information that accompanies the paper. The raw data itself should be archived (see above) rather than submitted to the journal as supplementary material.
- The Stage 2 manuscript must also contain a link to the registered protocol (deposited following AIP) in a standalone section entitled ‘Protocol Registration’.

# 14. Proceedings of the Royal Society B-Biological Sciences

<https://royalsocietypublishing.org/rspb/for-authors#question6>

## Open data in Proceedings B

We require supporting data and information, including source code and other digital research materials, to be made available at the time of submission so that that reviewers and Data Editors can assess your work and confirm that the archive is useful and complete. This is in line with our policies to promote greater openness in scientific research and to allow, as well as encourage, other researchers to perform full replications of published studies. For more information please refer to our data sharing policies (<https://royalsociety.org/journals/ethics-policies/data-sharing-mining/> ). In order to make it as easy as possible to comply with this policy, the Proceedings B submission system is fully integrated with the Dryad data repository. We also cover the cost of submitting data to Dryad.

The data, code and a comprehensive README file must be uploaded to a public data repository (such as Dryad, Zenodo, OSF or Figshare) and an anonymised link provided for reviewers and editors in your data statement. The README file must detail the contents of the data repository with metadata that allows readers to correctly interpret and understand the contents of the data files. At the submission stage, the deposit does not need to be made public, but please include a link that reviewers can access. Authors may upload data and code as supplementary material for review.

Datasets and code that have been deposited in an external repository should be appropriately cited in both the reference list and data accessibility section. Unless there are strong extenuating circumstances for doing so (e.g., genomic data that the authors wish to embargo), we will not accept statements such as "Data and materials are available upon request from the authors" in our data accessibility statements. The data, code or other digital research materials must be publicly accessible and clearly indicated as such, or must be publicly availably post embargo, and your manuscript will be returned to you in the event the Editor does not consider your data accessibility statement to meet our submission requirements, and you will be asked to provide further details. Exceptions to this policy are at the Editor’s discretion only.

**Data** Please include all data files used to generate a published result. Raw data (photos, videos, sound recordings, etc.) may also be included. Each file should be saved with a short, meaningful file name. Data files should be saved as comma-separated variable (.csv) files. For data in EXCEL spreadsheets, each worksheet of data should also be saved as a separate .csv file.

**Code** Thoroughly annotate your code with in-script comments indicating what the purpose of each set of commands is (i.e. “why?”). If the functioning of the code (i.e. “how”) is unclear, strongly consider re-writing it to be clearer/simpler.

**README file** Please include a README file (text (.txt) and Markdown (.md) are preferred) with information about your repository as a whole (code and files contents). Please include the following in the file:

- Title of the study
- Short summary of the study
- Code version (e.g., Git fingerprint, manual version number)
- Overview of folders/files and their contents
- Instructions for users to run the software (e.g. explain the project workflow and any configuration parameters of your software)
- Links to protocols.io or equivalent methods repositories, where applicable
- Once a paper is accepted, please include author names, contact details, links to preprint and the publication.

Double anonymous peer review (from January 2024) Datasets in repositories: please ensure that no identifying information are included in files deposited in any repositories. Files names should not include author names. If authors are depositing data in Dryad, please ensure that the README file does not contain any identifying information. For example: author line, grant IDs, project IDs, etc. Authors should not use first or last names when naming data files.

## Data sharing and mining

To allow others to verify and build on the work published in Royal Society journals, it is a condition of publication that authors make available the data, code and research materials supporting the results in the article. This policy can be cited by DOI via FAIRsharing.org. It is not permitted to state that data will be available from the authors upon request.

**Why do I need to make my data available?**

We require supporting data and information, including source code and other digital research materials, to be made publicly available on publication of articles, as well as at submission for verification/review purposes. This is in line with our policies to promote greater openness in scientific research. What are the benefits?

- It can increase citation levels and draw attention to your work
- Verification of results – readers can replicate studies and identify statistical or methodological errors
- Allow others to build on your work, find new uses for your data and use in meta-analyses (and reduce effort in data collection)
- Preserve your full scientific contributions (beyond published articles) in an organised, citable system
- Take advantage of professional curation services
- Providing data at submission means that accidental errors or problems with analysis may be picked up prior to publication

Learn more in our video (<https://www.youtube.com/watch?v=Ax7eptDTxY4> ).

**Where can I submit my data?**

There are two options for archiving data, code and other materials: in a publicly accessible repository (preferred) or as supplementary material in the published paper.

*Repositories*

Our preference is for authors to archive their raw data with an external repository, rather than providing this as supplementary material, since it will then be correctly curated. For example, a curated data repository will check that:

- All required materials have been received
- The data have no ethical, legal or rights issues which might prevent sharing
- The condition and format of the data are suitable for use and long-term preservation
- Documentation is sufficient to enable researchers to use the data

Authors should deposit research data in a FAIR-aligned repository, with a preference for those that explicitly follow the FAIR Data Principles and demonstrate compliance with international standards for data repositories (e.g. CoreTrustSeal).

Your chosen repository should:

- be publicly available
- retain data under an open license (CC0 or CC-BY) (clearly visible on the landing page of your dataset)
- provide files with a DOI
- make versioning/changes clear
- have provisions for permanent access
- have an English-language translation
- be curated

Use of Google drives, Dropbox, or similar services is prohibited for final publication but may be acceptable during the review process (check with the journal’s Editorial Office).

To encourage best practice in data sharing, several Royal Society journals have Dryad data deposition integrated into the journal submission system. For all its science journals, the Society will cover the cost ($120) of depositing data with Dryad. We have provided a list of other example repositories below – this list is not exhaustive; authors are encouraged to use the most appropriate repository for their field.

*Supplementary material*

Data files may alternatively be provided as a supplement to the paper, which we will host alongside the published article. In addition, we deposit all supplementary material into the Figshare repository on the author's behalf on publication. Our preference is that raw data is archived in an external repository (who will curate it properly as described above), and supplementary material is used for supporting figures, videos and other small files.

**Common repositories**

*General repositories*

Where no appropriate subject-specific repository exists, data should be deposited in a general repository such as Dryad or Zenodo.

**Biological Sciences**

*Nucleotide sequence data*

- Genbank
- European Molecular Biology Laboratory
- DNA Data Bank of Japan
- Accession numbers must be provided in the data accessibility section of your manuscript.
- Phylogenetic data
- TreeBASE

Please ensure that alignments as well as phylogenies are deposited.

*Microarray data*

- ArrayExpress
- Gene Expression Omnibus (GEO)
- Protein sequences
- Genbank
- European Molecular Biology Laboratory
- DNA Data Bank of Japan
- Protein Information Resource

Accession numbers must be provided in the data accessibility section of your manuscript.

*Proteomics data*

We recommend that all proteomics data, including mass spectrometry and protein interaction data is deposited via the EBI PRIDE website.

Physical Sciences

*Chemical data*

Chemical structures and bioassays should be deposited in PubChem.

*Earth, space and environmental science data*

A useful list of repositories can be found on the AGU website.

**What are the policies around code?**

Please provide access to all code used to generate statistics and generate figures, along with any (processed) data required as inputs, along with details of what software it requires (program and version). Analysis code (such as R scripts) must be made available at the point of submission, as well as any previously unreported algorithms. Any restrictions on or reasons for prohibiting the sharing of important code or algorithms must be discussed with the Editors before submission.

Source code should be made available under an open source licence and deposited in an appropriate repository such as Zenodo or Dryad. Small amounts of source code can be included in the supplementary material.

**When do I submit my data?**

Data files and other supporting material (such as details of code) must be provided at the point of submission for our Editors and reviewers for peer-review, and then made publicly available before publication. Files must be provided either by hosting them in an external repository with an accessible link included in the data accessibility section (you will be prompted for this during submission) or uploaded as supplementary material via the electronic submission system. For some of our journals, material may be provided via GitHub, Google drives, Dropbox, or similar services for the review stage, but they must be moved to a permanent, publicly accessible repository during revision. This must be finalised before the submission of a final version of the article.

**What level of data needs to be made available?**

It is a condition of publication that authors make the primary data, materials (such as statistical tools, protocols, software) and code publicly available. As a minimum, sufficient information and data are required to allow others to replicate all study findings reported in the article. Data and code should be deposited in a form that will allow maximum reuse. Studies that do not rely on data, code or other material (e.g., theoretical studies) to generate their conclusions, and so do not require data etc for replication attempts, may be exempt from our open data policy, but this must be clearly and explicitly stated in the cover letter and data access question in our online submission form.

All files, and all data columns within files, should be clearly labelled and readily interpretable. Provide a 'read-me' information file if necessary.

Authors do not need to submit the raw data collected during an investigation if the standard in the field is to share data that have been processed (e.g. CSV files recording response to stimuli rather than the electrical signals on which they were based). If processed data are supplied, rather than raw data, this should be stated in the data accessibility section during submission.

Raw image data for digital morphology should be provided with processed 3D data; e.g., modern field standards are to share such data in museum-linked repositories such as morphosource.org.

**What licence should apply to datasets?**

Please ensure that the licence applied to your dataset is clearly visible on the repository landing page of your data record. All data deposited to Dryad through the integrated submission system will be published under a Creative Commons BY 4.0 licence; as will all supplementary files. Data which do not explicitly have an open licence are not open data. Wherever possible, we ask that authors ensure that the license accompanying their data record is given as an open data license of either CC0 or CC-BY.

Exceptions to the above may be made for authors dependent on the circumstances (for example, due to ethical considerations, or if data are obtained from a third party where re-use restrictions may apply) but we ask that authors please query this with the editorial office prior to submission to the journal.

**How do I prepare the data accessibility section?**

Authors of all papers that report primary data will be required to provide a statement in the manuscript submission form that states where the article's supporting data, materials and code can be accessed.

If these have been deposited in an external repository this section should list the database, accession number/DOI and any other relevant details to clearly identify the dataset(s). Datasets included here must also be listed in the reference section. Citing datasets and code ensure effective and robust dissemination and appropriate credit to authors.

For example:

- DNA sequences: Genbank accessions F234391-F234402 [REF#]
- Phylogenetic data, including alignments: TreeBASE accession number S9123 [REF#]
- Climate data and MaxEnt input files: Dryad doi:10.5521/dryad.12311 [REF#]

If supporting data, materials or code have been included in the article’s supplementary material, this should be stated here, for example:

The datasets supporting this article have been uploaded as part of the supplementary material.

It is not permissible to state that data will be available upon request to the authors.

**How do I reference third party data or code in the data accessibility section?**

Please provide details about the previous published article, with a link where possible e.g. [X] data are available from Smith et al. [2021]: [URL XXX].

Where the third-party material isn't covered by an open licence, please provide evidence that you have permission to use the data.

**How do I cite datasets and code in the references?**

Citing datasets and code ensures effective and robust dissemination and appropriate credit to authors. Therefore, we strongly encourage authors to include datasets and code in the reference list as well as in your data accessibility section.

Citations in Royal Society journals are in the Vancouver style, for example:

1.Torres-Campos I, Abram PK, Guerra-Grenier E, Boivin G, Brodeur J. 2016 Data from: A scenario for the evolution of selective egg colouration: the roles of enemy-free space, camouflage, thermoregulation, and pigment limitation. Dryad Digital Repository https://doi.org/10.5061/dryad.5qt2k

Source code or the commercially available software used should be referenced in an appropriately formatted citation – this article (<https://doi.org/10.12688/f1000research.26932.2> ) provides guidance.

**What do I do if there are restrictions on accessing my data due to ethical and/or legal reasons?**

If data are restricted e.g., for ethical and/or legal reasons, you should make provisions for them to be available upon request to a Data Access Committee or Ethics Committee. In your data accessibility statement you should state the reason for restriction (e.g. identifiable patient data), the name of the Data Access Committee or Ethics Committee and details for the point of contact.

**What are the embargo restrictions?**

The general policy is that data, code and materials must be made publicly available at the time of publication. Exceptions to this policy are rare and can only be approved at the journal’s discretion. In some circumstances, embargoes on data sharing of up to one year may be granted.

# 15. Systematic Biology

<https://academic.oup.com/sysbio/pages/General_Instructions>

## Data and Supplementary Materials

### Data availability policy

All datasets used in the research for the manuscript must be made available to reviewers unless the data are already published elsewhere. Datasets also must be clearly described in the manuscript in the Data Availability Statement (see below).

For manuscripts involving phylogenetic analyses, electronic copies of data sets (e.g. nucleotide sequence data and new alignments of previously published data), in nexus format, must be supplied. Data files should also be provided for morphological analyses. Nucleotide/amino acid sequence data must be submitted to GenBank or EMBL. Genomic data can be submitted to NCBI Bioproject. Morphological data must be submitted to either Morphbank or MorphoBank. In all cases, data accession numbers must be provided in the text.

Alternative arrangements may be made for very large data files associated with studies using simulations.

### Data deposit process

At the time of submission, authors will be provided with a provisional Dryad link, which will allow editors and reviewers to access the online-only material but will not be made public. At the time of acceptance, a permanent Dryad link will be provided, allowing all every reader to access the data. Please ensure that the link is active. The first time you mention your dataset in the text of your paper, provide the DOI provided by Dryad as the location where the material can be found: either i) the provisional link during the different stages of the peer-review process, or ii) the permanent link once the paper is accepted and goes into production.

You may further choose to provide Supplementary Material with your article files, which will be available for download directly from your article page. Guidance on preparing and labeling these files is available on Oxford Academic's Preparing and submitting your manuscript webpage.

Check over your Online Appendices or Supplemental Material (if any) carefully, because they will not be copyedited or proofread, and cannot be changed later.

### Data Availability Statement

The inclusion of a Data Availability Statement is a requirement for articles published in Systematic Biology. Data Availability Statements provide a standardised format for readers to understand the availability of data underlying the research results described in the article. The statement may refer to original data generated in the course of the study or to third-party data analysed in the article. The statement should be written as its own section, after the Discussion or Conclusions, and before the Acknowledgements. The Data Availability Statement should describe and provide means of access, where possible, by linking to the data or providing the required unique identifier, for example:

Data files and/or online-only appendices can be found in the Dryad data repository: http://dx.doi.org/10.5061/dryad.[NNNN]

More information and example Data Availability statements can be found on Oxford Academic's Research Data webpage (<https://academic.oup.com/pages/open-research/research-data?login=true#data2> ).

### Dataset Citation

Systematic Biology supports the Force 11 Data Citation Principles and requires that all publicly available datasets be fully referenced in the reference list with an accession number or unique identifier such as a digital object identifier (DOI). Data citations should include the minimum information recommended by DataCite:

[dataset]* Authors, Year, Title, Publisher (repository name), Identifier.

*The inclusion of the [dataset] tag at the beginning of the citation helps us to correctly identify and tag the citation. This tag will be removed from the citation published in the reference list.

### Software for Systematics and Evolution Articles

Submissions should describe new or original software or tools that provide new analytical capabilities to the end user. Submissions may also be considered that describe new versions of existing software, provided that the new version makes significant changes to function or performance (for example, a version that implements new and important methods in addition to those previously provided in a software package might be considered for publication). Publication will be determined largely based on the software or tool itself, so working links to a functional copy must be provided at the time of submission.

Additional requirements: The software or tool must well documented and easy to use for the typical user. The manuscript itself must be readable by the general Systematic Biology readership. If relevant, the manuscript must include benchmark data, or refer to Supplemental Material that includes such data. If appropriate, such benchmarking should include real biological data and a comparison with related tools. If appropriate, the submission must include working sample data files. Any software must be open source, web-distributed and free to non-commercial users. In addition, the authors must certify that they will provide support for the software or tools for a minimum of two years from the date of publication. Systematic Biology encourages the use of GPL-like licenses and the use of open repositories, such as SourceForge or Google Code.

Software for Systematics and Evolution papers should include an abstract. We will not enforce any specific organization of the text, but the following suggestions might help in organizing a submission: an introduction that describes the motivation; a Description section; a Benchmark section; a Biological Examples section (if applicable); a statement regarding Availability. However the manuscript is organized, please pay careful attention to the normal formatting for section headings, references, and other aspects of the journal’s style.

# 16. The ISME Journal

<https://www.nature.com/ismej/authors-and-referees/data-availability-and-policy>

## Data Availability and Policy

The ISME Journal adheres to Springer Nature’s Data Policy Type 3. This means that a submission to The ISME Journal implies that materials described in the manuscript, including all relevant raw data, will be freely available to any researcher wishing to use them for non-commercial purposes, without breaching participant confidentiality. It also means that a Data Availability Statement (see below for more details) is required by the journal.

Data Policy Details

The journal strongly encourages that all datasets on which the conclusions of the paper rely should be available to readers. We encourage authors to ensure that their datasets are either deposited in publicly available repositories (where available and appropriate) or presented in the main manuscript or additional supporting files whenever possible. Please see Springer Nature’s information on recommended repositories.

- List of Repositories (<https://www.springernature.com/la/authors/research-data-policy/recommended-repositories> )
- Research Data Policy (<https://www.springernature.com/la/authors/research-data-policy/data-policy-faqs> )

General repositories – for all types of research data – such as figshare and Dryad may be used where appropriate.

Where a widely established research community expectation for data archiving in public repositories exists, submission to a community-endorsed, public repository is mandatory*.

Persistent identifiers (such as DOIs and accession numbers) for relevant datasets must be provided in the paper.

*For the following types of data set, submission to a community-endorsed, public repository is mandatory:


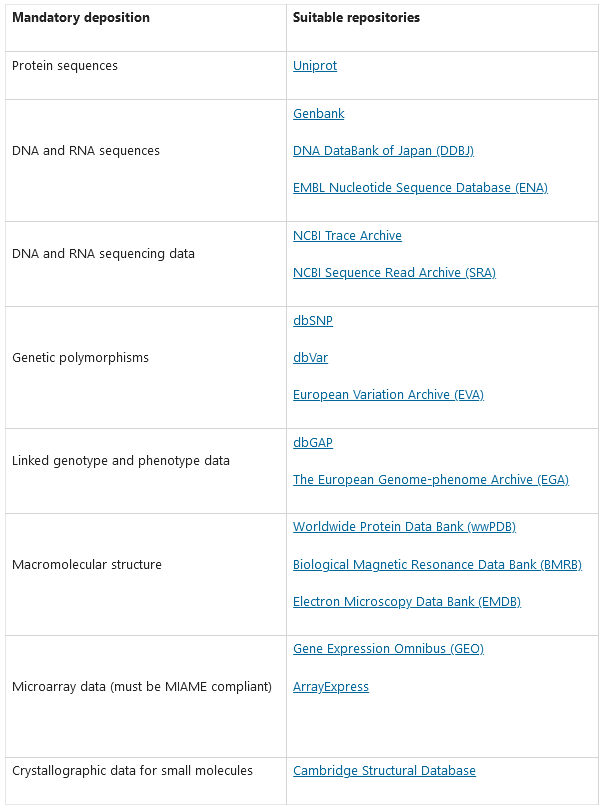


### Data Availability Statement

As part of the The ISME Journal Data Availability Policies, all original articles must include a Data Availability Statement. Data availability statements should include information on where data supporting the results reported in the article can be found including, where applicable, hyperlinks to publicly archived datasets analysed or generated during the study. By data we mean the minimal dataset that would be necessary to interpret, replicate and build upon the findings reported in the article. We recognise it is not always possible to share research data publicly, for instance when individual privacy could be compromised, and in such instances data availability should still be stated in the manuscript along with any conditions for access.

Data Availability statements can take one of the following forms (or a combination of more than one if required for multiple datasets):

- The datasets generated during and/or analysed during the current study are available in the [NAME] repository, [PERSISTENT WEB LINK TO DATASETS].
- The datasets generated during and/or analysed during the current study are not publicly available due [REASON WHY DATA ARE NOT PUBLIC] but are available from the corresponding author on reasonable request.
- The datasets generated during and/or analysed during the current study are available from the corresponding author on reasonable request.
- Data sharing not applicable to this article as no datasets were generated or analysed during the current study.
- All data generated or analysed during this study are included in this published article [and its supplementary information files].
- The data that support the findings of this study are available from [third party name] but restrictions apply to the availability of these data, which were used under license for the current study, and so are not publicly available. Data are however available from the authors upon reasonable request and with permission of [third party name].

<https://www.nature.com/ismej/authors-and-referees/gta>

**Data Availability Statement**: An inherent principle of publication is that others should be able to replicate and build upon the authors' published claims. The ISME Journal adheres to Springer Nature’s Data Policy Type 3 (<https://www.springernature.com/gp/authors/research-data-policy/research-data-policy-types> ).

This means that a submission to the journal implies that materials described in the manuscript, including all relevant raw data, will be freely available to any researcher wishing to use them for non-commercial purposes, without breaching participant confidentiality. It also means that a Data Availability Statement (see here <https://www.nature.com/ismej/authors-and-referees/data-availability-and-policy> for more details) must be included as part of your manuscript.

**Legacy data policy types**

All Springer Nature journals are moving to a policy that requires data availability statements for primary research articles. This is already in place for BMC, Nature and SpringerOpen titles and is being progressively adopted by Springer and Palgrave Macmillan journals.

While the implementation is underway, certain Springer and Palgrave Macmillan journals will retain the older data policy types, as outlined below. The specific data policy of each journal is stated in the submission guidance. ‘Type 3’ is equivalent to our new, standardised data policy.


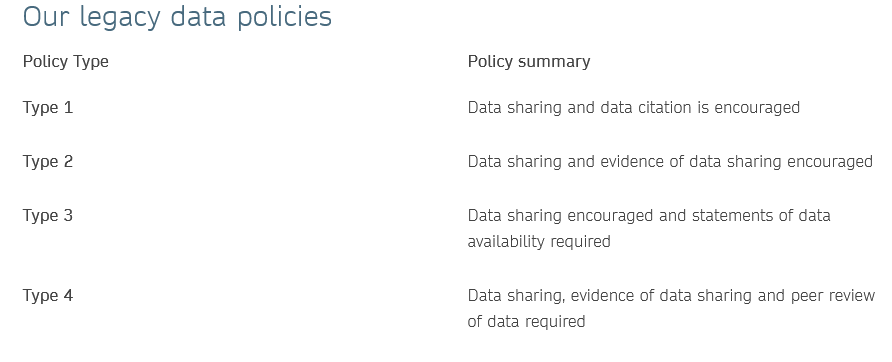


<https://www.springernature.com/gp/authors/research-data-policy>

## Research data policy

At Springer Nature we advance discovery by publishing trusted research, supporting the development of new ideas and championing open science. We also aim to facilitate compliance with research funder and institution requirements to share data.

To help accomplish this we have established a standard research data policy for our journals, based on transparency around supporting data. This policy applies to all datasets that are necessary to interpret and replicate the conclusions reported in a research article.

**1. All original articles must include a data availability statement**

Data availability statements should include information on what data are available, where these can be found, and any applicable access terms. This applies to both original and reused data, and whether or not data can be shared publicly. See our guidance on data availability statements for more information (<https://www.springernature.com/gp/authors/research-data-policy/data-availability-statements> ).

**2. We strongly encourage that all datasets supporting the analysis and conclusions of the paper are made publicly available at the time of publication, and we mandate the sharing of community-endorsed data types**

We encourage authors to deposit their supporting data in publicly available repositories, or failing this within the manuscript or additional supporting files. See our repository guidance for more information (<https://www.springernature.com/gp/authors/research-data-policy/recommended-repositories> ).

For a number of data types, submission to a community-endorsed, public repository is mandatory. See our list of mandated data types (<https://www.springernature.com/gp/authors/research-data-policy/repositories-mandates/19540364> ).

**3. Peer reviewers are entitled to request access to underlying data (and code) when needed to perform their evaluation of a manuscript.**

**4. We recognise it is not always possible to share research data publicly, for instance when privacy of research participants could be compromised. In such instances data availability should still be stated in the manuscript along with any conditions for access.**

A large number of our journals already support this policy, including Nature Portfolio, BMC and many Springer and Palgrave titles. We are in the process of implementing this policy across the remainder of our portfolio in stages.

For information on a journal’s specific policies, please consult the journal submission guidelines.

# 17. Trends in Ecology and Evolution

<https://www.cell.com/trends/ecology-evolution/authors>

Please note that all articles in TREE are peer reviewed, and publication cannot be guaranteed. TREE is not a primary publication and thus cannot consider manuscripts that rely on unpublished data.
